# The utility of mtDNA and rDNA for barcoding and phylogeny of plant-parasitic nematodes from Longidoridae (Nematoda, Enoplea)

**DOI:** 10.1038/s41598-017-11085-4

**Published:** 2017-09-07

**Authors:** J. E. Palomares-Rius, C. Cantalapiedra-Navarrete, A. Archidona-Yuste, S. A. Subbotin, P. Castillo

**Affiliations:** 1 Instituto de Agricultura Sostenible (IAS), Agencia Estatal Consejo Superior de Investigaciones Científicas (CSIC), Avda. Menéndez Pidal s/n, 14004 Córdoba, Campus de Excelencia Internacional Agroalimentario, ceiA3 Spain; 2 Plant Pest Diagnostic Center, California Department of Food and Agriculture, 3294 Meadowview Road, Sacramento, CA 95832-1448 USA; 3 Center of Parasitology of A.N. Severtsov Institute of Ecology and Evolution of the Russian Academy of Sciences, Leninskii Prospect 33, Moscow, 117071 Russia

## Abstract

The traditional identification of plant-parasitic nematode species by morphology and morphometric studies is very difficult because of high morphological variability that can lead to considerable overlap of many characteristics and their ambiguous interpretation. For this reason, it is essential to implement approaches to ensure accurate species identification. DNA barcoding aids in identification and advances species discovery. This study sought to unravel the use of the mitochondrial marker cytochrome c oxidase subunit 1 (*coxI*) as barcode for Longidoridae species identification, and as a phylogenetic marker. The results showed that mitochondrial and ribosomal markers could be used as barcoding markers, except for some species from the *Xiphinema americanum* group. The ITS1 region showed a promising role in barcoding for species identification because of the clear molecular variability among species. Some species presented important molecular variability in *coxI*. The analysis of the newly provided sequences and the sequences deposited in GenBank showed plausible misidentifications, and the use of voucher species and topotype specimens is a priority for this group of nematodes. The use of *coxI* and D2 and D3 expansion segments of the 28S rRNA gene did not clarify the phylogeny at the genus level.

## Introduction

The phylum Nematoda comprises one of the largest and most diverse groups of animals. Most species are found in oceanic, freshwater and soil ecosystems, and only a few are pathogens of animals and plants^1^. Plant-parasitic nematodes (PPNs) have a diverse morphology and parasitic habits^2^. PPNs are distributed between the classes Chromadorea and Enoplea within very restricted orders (Rhabditida, Dorylaimida and Triplonchida)^3^. The order Dorylaimida, which belongs to Enoplea, includes several genera of PPNs in the family Longidoridae (*Australodorus*, *Longidoroides*, *Longidorus*, *Paralongidorus*, *Paraxiphidorus*, *Xiphidorus* and *Xiphinema*)^3^. These nematodes are of particular scientific and economic interest because they directly damage the roots of the host plant and some are vectors of several plant viruses (genus *Nepovirus*) that cause severe damage to a wide variety of crops^4^. Because of its great morphological diversity, the genus *Xiphinema* has been divided into two species groups^5–8^: (*i*) the *Xiphinema americanum* group, which comprises a complex of approximately 60 species, and (*ii*) the *Xiphinema* non*-americanum* group, which comprises a complex of more than 200 species. The traditional identification of these species by morphology and morphometric studies is very difficult because of their high intra-specific morphological variability, which can lead to considerable overlap of many characteristics and ambiguous interpretation^6, 9^. For this reason, new approaches are needed to ensure accurate species identification. Recently, numerous species from Longidoridae (44.4%) were molecularly characterized by ribosomal RNA genes (rDNA), i.e. partial 18S, ITS regions, or the D2 and D3 expansion segments of the 28S rRNA gene, as well as by the protein-coding mitochondrial gene cytochrome c oxidase subunit 1 (*coxI*), constituting a useful tool for species identification and the establishment of phylogenetic relationships within PPNs^6, 10–14^. Several studies conducted with 18S rRNA gene sequences^11, 15, 16^ did not provide taxonomic clarity among Longidoridae, since this gene seems to evolve too slowly to be useful as an appropriate marker for phylogenetic studies at the species level. The ITS region, D2–D3 of 28S rDNA sequences, and the *coxI* gene could be considered good markers for species identification. However, due to molecular variability in the ITS region, it appears better suited for species identification than for phylogenetic analysis^17^. Additionally, recent studies showed that mtDNA genes evolve much more quickly than rRNA genes, revealing low intra-specific and high inter-specific molecular variability for Longidoridae^12, 16, 18–21^. Therefore, it seems to be the most promising marker to relieve taxonomic confusion within this group. The *coxI* gene is frequently used as an efficient marker for species identification in the animal kingdom and may also be used to estimate species richness, particularly in understudied faunas^22^.

Therefore, the objectives of this research were to evaluate the variability of the mitochondrial marker gene *coxI* and partial sequence of the 28S rRNA gene within Longidoridae, as well as their usefulness as markers for barcoding and for reconstructing the phylogeny of the group.

## Results and Discussion

### *coxI* amplification in Longidoridae

A total of 136 new accessions belonging to 82 species for *coxI* were obtained for the first time in this study (Tables 1 and S1). Taxon coverage (species/genus species) of 11.9%, 8.3%, and 1.5% was achieved for *Xiphinema*, *Longidorus* and *Paralongidorus*, respectively. PCR amplification and sequencing for the partial *coxI* were carried out by combining several primers (Table 1). The best set of primers were COIF/XIPHR2^21^, followed by JB3/JB4^23^, COIF/COIR and COIF/XIPHR1^21^. These sets of primers amplified a single fragment of approximately 500 bp. We did not find amplification of pseudogenes using these sets of primers. However, we did not perform a systematic analysis of primer amplification, as we started with the combination COIF/XIPHR2 in the majority of the studied samples; this combination was reported to be efficient in previous studies^21^. All new partial *coxI* sequences were obtained using voucher specimens identified by integrative taxonomy, with the combination of morphological characteristics and unequivocal molecular markers from the same individual nematode, *viz*. the D2–D3 region (Tables 1 and S1) and ITS1 in some cases.

**Table 1:** Taxa sampled for dagger and needle nematodes species of the family Longidoridae and sequences of cytochrome *c* oxidase subunit 1 (*coxI*) used in this study. Species identifications were based on morphology and barcoding using D2–D3 expansion segments of 28S rDNA^1^.

Nematode species	Sample code	Locality	Host plant	GenBank accession numbers	
				*28S*	*coxI*
**Genus** ***Xiphinema***					
1.*Xiphinema adenohystherum*	SORIA	Arévalo de la Sierra, Soria province, Spain	european holly	KC567164	KY816588
*Xiphinema adenohystherum*	ALMAG	Almagro, Ciudad Real province, Spain	wild olive	*^2^	KY816589
*Xiphinema adenohystherum*	AR086	Prado del Rey, Cádiz province, Spain	wild olive	*	KY816590
*Xiphinema adenohystherum*	AR078	Almodóvar, Córdoba province, Spain	wild olive	*	KY816591
*Xiphinema adenohystherum*	IASNB	Jerez de la Frontera, Cádiz province, Spain	wild olive	*	KY816592
2.*Xiphinema andalusiense*	ARO93	Belmez,Córdoba, Spain	wild olive	KX244884	KY816593
*Xiphinema andalusiense*	00419	Andújar, Jaén, Spain	wild olive	KX244885	KY816594
*Xiphinema andalusiense*	AR108	Villaviciosa de Córdoba, Córdoba, Spain	wild olive	KX244888	KY816595
3.*Xiphinema baetica*	LOMAS	Hinojos, Huelva province, Spain	stone pine	KC567165	KY816596
*Xiphinema baetica*	HATRA	Villamanrique de la Condesa, Huelva, Spain	cork oak	KC567166	KY816597
4.*Xiphinema belmontense*	MOUCH	Merza, Pontevedra province, Spain	chestnut	KC567171	KY816598
5.*Xiphinema cadavalense*	ST077	Espiel, Córdoba province, Spain	cultivated olive	KX244932	KY816599
6.*Xiphinema celtiense*	AR083	Adamuz, Córdoba province, Spain	wild olive	KX244889	KY816600
*Xiphinema celtiense*	AR082	Adamuz, Córdoba province, Spain	wild olive	KX244890	KY816601
7.*Xiphinema cohni*	J0126	Puerto de Sta. María, Cádiz province, Spain	grapevine	KC567173	KY816602
8.*Xiphinema conurum*	ST45V	Sorbas, Almería province, Spain	cultivated olive	KX244892	KY816603
9.*Xiphinema costaricense*	ACC86	Guayabo, Turrialba, Cartago, Costa Rica	forest	KX931056	KY816604
*Xiphinema costaricense*	ACC46	Santa Rosa, Limón, Limón	cocoa	KX931057	KY816605
10 *Xiphinema coxi europaeum*	AR020	Hinojos, Huelva province, Spain	wild olive	KC567174	KY816606
*Xiphinema coxi europaeum*	H0027	Almonte, Huelva province, Spain	cork oak	KC567177	KY816607
11.*Xiphinema cretense*	AR039	Hersonisos province, Crete, Greece	wild olive	KJ802878	KY816608
12.*Xiphinema duriense* ^3^	ST02C	Gibraleón, Huelva province, Spain	cultivated olive	KP268963	KY816609
13.*Xiphinema gersoni*	H0059	Almonte, Huelva province, Spain	eucalyptus	KC567180	KY816610
14.*Xiphinema herakliense*	OLEA8	Vathy Rema, Crete, Greece	wild olive	KM586345	KY816611
*Xiphinema herakliense*	OLEA17	Agiofarago, Crete, Greece	wild olive	KM586346	KY816612
*Xiphinema herakliense*	OLE18	Agiofarago, Crete, Greece	wild olive	KM58634 9	KY816613
15.*Xiphinema hispanum*	00419	Andújar, Jaén province, Spain	wild olive	GU725074	KY816614
16.*Xiphinema hispidum*	AR098	Bollullos par del Condado, Huelva province, Spain	grapevine	KC567181	KY816615
*Xiphinema hispidum*	H0026	Rociana del Condado, Huelva province, Spain	grapevine	HM921366	KY816616
17.*Xiphinema insigne*	MIYA1	Miyazaki, Japan	*Prunus* sp.	*	KY816617
18.*Xiphinema israeliae*	AR013	Roufas province, Greece	wild olive	KJ802883	KY816618
19.*Xiphinema italiae*	AR041	Las Tres Villas, Almería province, Spain	wild olive	KX244911	KY816619
*Xiphinema italiae*	AR091	Puerto Real, Cádiz province, Spain	wild olive	KX244912	KY816620
*Xiphinema italiae*	TUNIS	Sbitla, Kasserine, Tunisia	cultivated olive	KX062674	KY816621
*Xiphinema italiae*	TUN11	Sbiba, Kasserine, Tunisia	cultivated olive	KX062677	KY816622
*Xiphinema italiae*	APUL	Bari, Bari province, Italy	grapevine	*	KY816623
20.*Xiphinema iznajarense*	JAO25	Iznájar, Córdoba province, Spain	cultivated olive	KX244892	KY816624
21.*Xiphinema krugi*	ACC47	Sucre, Ciudad Quesada, Alajuela, Costa Rica	Robust star-grass	KX931061	KY816625
*Xiphinema krugi*	ACC13	Santa Gertrudis, Grecia, Alajuela, Costa Rica	Sugar-cane	KX931060	KY816626
22.*Xiphinema luci*	IAGRQ	Benacazón, Sevilla province, Spain	rose	KP268965	KY816627
23.*Xiphinema lupini*	H0050	Hinojos, Huelva province, Spain	grapevine	KC567183	KY816628
*Xiphinema lupini*	388GD	Bollullos par del Condado, Huelva province, Spain	grapevine	HM921352	KY816629
*Xiphinema lupini*	388GD	Bollullos par del Condado, Huelva province, Spain	grapevine	*	KY816630
24.*Xiphinema macroacanthum*	ITAL	Brindisi province, Italy	cultivated olive	*	KY816631
25.*Xiphinema macrodora*	AR097	Santa Mª de Trassierra, Córdoba province, Spain	wild olive	KU171044	KY816632
26.*Xiphinema mengibarense*	O3C04	Mengíbar, Jaen province, Spain	cultivated olive	KX244893	KY816633
*Xiphinema mengibarense*	O30V5	Mengíbar, Jaen province, Spain	cultivated olive	KX244894	KY816634
27.*Xiphinema meridianum*	11R16	Sbitla, Kasserine, Tunisia	cultivated olive	KX062678	KY816635
28.*Xiphinema nuragicum*	ST012	Espejo, Córdoba province, Spain	grapevine	*	KY816636
*Xiphinema nuragicum*	AR054	Medina Sidonia, Cádiz province, Spain	wild olive	*	KY816637
*Xiphinema nuragicum*	ST106	La Puebla de los Infantes, Sevilla province, Spain	cultivated olive	*	KY816638
*Xiphinema nuragicum*	JAO28	Antequera, Málaga province, Spain	cultivated olive	*	KY816639
*Xiphinema nuragicum*	AR113	Alcolea, Córdoba province, Spain	wild olive	*	KY816640
29.*Xiphinema opisthohysterum*	AR031	Tarifa, Cádiz province, Spain	wild olive	KP268967	KY816641
*Xiphinema opisthohysterum*	00418	Andújar, Jaén province, Spain	grasses	JQ990040	KY816642
30.*Xiphinema pseudocoxi*	AR095	Alcaracejos, Córdoba province, Spain	wild olive	KX244915	KY816643
31.*Xiphinema pyrenaicum*	ESMEN	Cahors, Quercy province, France	grapevine	GU725073	KY816644
32.*Xiphinema rivesi*	CASLO	Castillo de Locubín, Jaén province, Spain	cherry tree	JQ990037	KY816645
*Xiphinema rivesi*	00518	Moriles, Córdoba province, Spain	grapevine	HM921357	KY816646
33.*Xiphinema robbinsi*	12R28	Sbitla, Kasserine, Tunisia	cultivated olive	KX062683	KY816647
34.*Xiphinema setariae*	ACC09	Pueblo Nuevo de Duacarí, Limón, Costa Rica	banana	KX931066	KY816648
35.*Xiphinema sphaerocephalum*	AR063	Coto Ríos, Jaén province, Spain	wild olive	*	KY816649
36.*Xiphinema turcicum*	ST149	San José del Valle, Cádiz province, Spain	wild olive	*	KY816650
37.*Xiphinema turdetanense*	AR0015	Sanlúcar de Barrameda, Cádiz province, Spain	wild olive	KC567186	KY816651
38.*Xiphinema vallense*	AR0027	Bolonia, Cádiz province, Spain	wild olive	KP268960	KY816652
*Xiphinema vallense*	H00003	Hinojos, Huelva province, Spain	cultivated olive	KP268961	KY816653
39.*Xiphinema* sp.	P0011	Sbitla, Kasserine, Tunisia	cultivated olive	KX062686	KY816654
**Genus** ***Longidorus***					
40.*Longidorus aetnaeus*	CD1138	Varenikovskaya, Krymsk, Krasnodar Terr., Russia	silver poplar	KF242324	KY816655
*Longidorus aetnaeus*	CD1108	Varenikovskaya, Krymsk, Krasnodar Terr., Russia	*Populus* sp.	KF242323	KY816656
*Longidorus aetnaeus*	CD1111	Varenikovskaya, Krymsk, Krasnodar Terr., Russia	*Salix fragilis*	KF242318	KY816657
*Longidorus aetnaeus*	CD1129	Varenikovskaya, Krymsk, Krasnodar Terr., Russia	*Acer tataricum*	KF242321	KY816658
*Longidorus aetnaeus*	CD1143	Varenikovskaya, Krymsk, Krasnodar Terr., Russia	*Salix alba*	KF242322	KY816659
41.*Longidorus africanus*	P00011	Chott-mariem province, Tunisia	cultivated olive	KX062665	KY816660
42.*Longidorus alvegus*	ALNOR	Andújar, Jaén province, Spain	black alder	KT308867	KY816661
43.*Longidorus andalusicus*	J0172	Sanlúcar de Barrameda, Cádiz province, Spain	pickle weed	JX445118	KY816662
44.*Longidorus apulus*	BARLE	Barletta, Bari province, Italy	artichoke	AY601571	KY816663
45.*Longidorus artemisiae*	CD1127	Shestikhino, Myshkin district, Yaroslavl, Russia	*Poa* sp.	KF242314	KY816664
46.*Longidorus asiaticus*	LARGE	Bari province, Italy	crape myrtle	KR351254	KY816665
47.*Longidorus baeticus*	M0121	Montemayor, Córdoba province, Spain	grapevine	JX445106	KY816666
48.*Longidorus closelongatus*	23CRE	Mires, Heraklion province, Crete, Greece	grapevine	KJ802865	KY816667
49.*Longidorus crataegi*	M0156	Montemayor, Córdoba province, Spain	grapevine	JX445114	KY816668
*Longidorus crataegi*	M0156	Montemayor, Córdoba province, Spain	grapevine	*	KY816669
50.*Longidorus cretensis*	TOCRE	Pentamodi, Heraklion province, Crete, Greece	cultivated olive	KJ802868	KY816670
51.*Longidorus distinctus*	CD1128	Pyatigorsk, Stavropol Territory, Russia	*Salix sp*.	KF242317	KY816671
52.*Longidorus euonymus*	CD1118	Bolshoy Vyas, Lunino district, Russia	*Asparagus cicer*	KF242333	KY816672
*Longidorus euonymus*	CD1130	Anapa, Anapa district, Krasnodar Territory, Russia	*Juglans regia*	KF242332	KY816673
53.*Longidorus fasciatus*	M0063	Monturque, Córdoba province, Spain	grapevine	JX445108	KY816674
54.*Longidorus indalus*	ST042	Las Tres Villas, Almería province, Spain	cultivated olive	KT308854	KY816675
55.*Longidorus intermedius*	CD1122	Kamennomostsky, Adygeya, Russia	*Fagus orientalis*	KF242312	KY816676
56.*Longidorus iranicus*	GRECD	Harakas province, Crete, Greece	grapevine	KJ802875	KY816677
57.*Longidorus iuglandis*	H0183	Bonares, Huelva province, Spain	grapevine	JX445104	KY816678
58.*Longidorus jonesi*	MIY03	Miyazaki, Japan	*Prunus* sp.	KF552069	KY816679
59.*Longidorus kuiperi*	BOLOI	Bolonia, Cádiz province, Spain	marram grass	*	KY816680
60.*Longidorus laevicapitatus*	ACC01	La Virgen de Sarapiquí, Heredia, Costa Rica	Sugar cane	KX136865	KY816681
61.*Longidorus leptocephalus*	CD1119	Potrosovo, Kozelsk district, Kaluga region, Russia	common nettle	KF242326	KY816682
62.*Longidorus lignosus*	CD1120	Sukko, Anapa district, Krasnodar Territory, Russia	*Acer campestre*	KF242345	KY816683
63.*Longidorus lusitanicus*	J0212	Sanlúcar de Barrameda, Cádiz province, Spain	wild olive	KT308869	KY816684
64.*Longidorus macrodorus*	JAO06	La Grajuela, Córdoba province, Spain	cultivated olive	KT308855	KY816685
*Longidorus macrodorus*	JAO06	La Grajuela, Córdoba province, Spain	cultivated olive	KT308856	KY816686
65.*Longidorus magnus*	M0130	Aguilar de la Frontera, Córdoba province, Spain	cultivated olive	*	KY816687
*Longidorus magnus*	M0017	Lucena, Córdoba province, Spain	grapevine	JX445113	KY816688
*Longidorus magnus*	M0079	Monturque, Córdoba province, Spain	grapevine	*	KY816689
*Longidorus magnus*	J0164	Jerez de la Frontera, Cádiz province, Spain	grapevine	*	KY816690
*Longidorus magnus*	ST077	Espiel, Córdoba province, Spain	cultivated olive	*	KY816691
*Longidorus magnus*	JAO01	Villaviciosa de Córdoba, Córdoba province, Spain	cultivated olive	*	KY816692
*Longidorus magnus*	JAO31	Antequera, Málaga province, Spain	cultivated olive	*	KY816693
*Longidorus magnus*	CASLO	Castillo de Locubin, Jaén province, Spain.	cherry tree	*	KY816694
66.*Longidorus onubensis*	ST005	Niebla, Huelva province, Spain	cultivated olive	KT308857	KY816695
67.*Longidorus persicus*	ESMAE	Gilan-e-Gharb, Kermanshah province, Iran	rose	KT149799	KY816696
68.*Longidorus pisi*	0IRAN	Markazi province, Iran	apple tree	JQ240274	KY816697
69.*Longidorus pseudoelongatus*	AR034	Voutes province,Crete, Greece	cultivated olive	KJ802870	KY816698
*Longidorus pseudoelongatus*	AR040	Hersonisos province, Crete, Greece	cultivated olive	KJ802871	KY816699
70.*Longidorus rubi*	H0026	Almonte, Huelva province, Spain	*Pinus pinea*	JX445116	KY816700
71.*Longidorus silvestris*	AR027	Bolonia, Cádiz province, Spain	cultivated olive	KT308859	KY816701
72.*Longidorus vallensis*	AR055	San José del Valle, Cádiz province, Spain	wild olive	KT308861	KY816702
*Longidorus vallensis*	M0012	Cabra, Córdoba province, Spain	grapevine	KT308862	KY816703
73.*Longidorus vineacola*	AR031	Tarifa, Cádiz province, Spain	wild olive	KT308873	KY816704
*Longidorus vineacola*	AR113	Alcolea, Córdoba province, Spain	wild olive	*	KY816705
*Longidorus vineacola*	TRASI	Santa Mª de Trassierra, Córdoba province, Spain	cultivated olive	*	KY816706
*Longidorus vineacola*	M0124	Montemayor, Córdoba province, Spain	Portuguese oak	*	KY816707
*Longidorus vineacola*	M0124	Montemayor, Córdoba province, Spain	Portuguese oak	*	KY816708
*Longidorus vineacola*	0419B	Andújar, Jaen province, Spain	wild olive	*	KY816709
*Longidorus vineacola*	H0089	Almonte, Huelva province, Spain	Stone pine	*	KY816710
*Longidorus vineacola*	ST117	Setenil de las Bodegas, Cádiz province, Spain	cultivated olive	*	KY816711
*Longidorus vineacola*	ST016	El Saucejo, Sevilla province, Spain	cultivated olive	KT308872	KY816712
74.*Longidorus vinearum*	AR097	Santa Mª de Trassierra, Córdoba province, Spain	wild olive	KT308876	KY816713
75.*Longidorus wicuolea*	AR0101	Bonares, Huelva province, Spain	wild olive	KT308865	KY816714
76.*Longidorus* sp.3	CD1112	Natukhaevskaya, Krasnodar Territory, Russia	*Prunus divaricata*	KF242335	KY816715
77.*Longidorus* sp.4	CD1117	Proletarka, Krasnosulinsk, Rostov region, Russia	*Salix babylonica*	KF242334	KY816716
78.*Longidorus* sp.6	CD876	Point Reyes, Marin county, California, USA	unknown	KF242328	KY816717
**Genus** ***Paralongidorus***					
79.*Paralongidorus bikanerensis*	BAMIR	Bam, Kerman province, Iran	Palm	JN032584	KY816718
80.*Paralongidorus iranicus*	NOURI	Nour, Mazandaran province, Iran	Pine	JN032587	KY816719
81.*Paralongidorus litoralis*	ZAHAR	Zahara de los Atunes, Cádiz province, Spain	mask tree	EU026155	KY816720
82.*Paralongidorus paramaximus*	ALGUC	Alcalá de Guadaira, Sevilla province, Spain	citrus	EU026156	KY816721
*Paralongidorus paramaximus*	ALGUC	Alcalá de Guadaira, Sevilla province, Spain	citrus	*	KY816722
*Paralongidorus paramaximus*	ALGUC	Alcalá de Guadaira, Sevilla province, Spain	citrus	*	KY816723

^1^For species identification see refs 9, 19, 20, 25, 27, 39, 40, 43–47, 63–69. ^2^(*) Sequenced population but not deposited in GenBank database, since was identical to other sequences of the same species already deposited in GenBank. ^3^The previous Accession JQ990053 reported as belonging to *X. duriense* was a mistake, and has been already corrected in NCBI, and replaced here by the correct one (accurately sequenced from the same specimen than D2–D3) and replaced by the new correct sequence KY816609 in this study.

### mtDNA and rDNA molecular variability

To our knowledge, the present study is the largest survey ever conducted for Longidoridae mtDNA and rDNA molecular variability. It covers 44 species (268 sequences), 112 species (577 sequences) and 64 species (252 sequences) for partial *coxI*, D2–D3 and ITS respectively, with more than one sequence per species as available in GenBank or obtained in this study (Tables S2–S4). However, some genera of Longidoridae were underrepresented (*e.g., Paralongidorus* and *Xiphidorus*) (Table S1).

For the partial *coxI* gene, 14 species (101 sequences) from the *X. americanum* group were studied, of which 7 showed a percent similarity lower than 90%: *X. americanum* (78.82%), *X. brevicolle* ‘complex’ (76.67%), *X. californicum* (89.83%), *X. incognitum* (86.61%), *X. rivesi* (70.94%), *X. peruvianum* (79.71%) and *Xiphinema* sp. 1 (82.66%). In the *X*. non-*americanum* group, intra-specific molecular variability of *coxI* was analysed in 18 species (89 sequences), but only two species within this group showed similarity values lower than 90%: *X. adenohystherum* (88.40%) and *X. italiae* (69.73%). The intra-specific molecular variability detected in 11 studied *Longidorus* species (52 sequences) was high; 4 of them showed a percentage of similarity below 85%: *L. magnus* (78.70%), *L. orientalis* (78.78%), *L. poessneckensis* (84.62%), and *L. vineacola* (68.91%). Finally, only one species from the genus *Paralongidorus* with available partial *coxI* sequences was found—*Paralongidorus paramaximus*—with 99% similarity between the three sequences analysed.

The majority of sequence variability in all the studied genera appears at the third codon position, as for *L. helveticus*, which showed a sequence similarity of 92.66% with all variations at silent sites^24^, or *L. poessneckensis*, which showed an 81% sequence similarity with all molecular variability at silent sites, except for two nucleotides that caused changes in the amino acid sequence^25^. In the majority of the studied cases, mean Kimura 2-parameter distance (K2P) values did not exceed the interspecific distance mean, except for 5 species from the *X. americanum* group: *X. americanum*, *X. brevicolle* ‘complex’, *X. peruvianum*, *X. rivesi*, and *Xiphinema* sp. 1. However, these species comprise species complexes that must be further studied, as recently proposed by Orlando *et al*., because some of them may have been misidentified^26^. In contrast, intra-specific molecular variability detected in *X. italiae* and *X. adenohystherum* was accurate and correct. In both cases, these species were identified by integrative taxonomic approaches, and molecular analyses were performed using the same DNA extraction of single individuals for different markers (D2–D3 and *coxI*). Integrative identification of the *X*. non-*americanum* group is apparently less difficult due to more taxonomically informative traits (*e.g*., uterine differentiation) and the higher number of species molecularly studied. Similarly, *Longidorus* spp. with higher intra-specific variability were clearly delineated in this study (*viz. L. vineacola* and *L. magnus*) and previous studies (*viz. L. orientalis* ^27^, *L. poessneckensis* ^25^ and *L. helveticus* ^24^), using integrative taxonomy and the combination of unequivocal molecular markers (D2–D3 and partial *coxI*) from single individuals. Our results suggest that intra-specific variation in the partial *coxI* gene may be higher than expected. However, more species and more populations should be studied in the future to clarify the real molecular variability among species within Longidoridae.

In contrast, the D2–D3 region showed low intra-specific molecular variability, since no similarity value below 95% was detected for any of the studied species (except *X. americanum*, with 94.65% similarity), even though there are more sequences from this region than for the partial *coxI* (112 species for D2–D3 *vs* 43 species for *coxI*) (Table S3). However, this lower intra-specific molecular variability may confound species identification, especially within the *X. americanum* group, where seven species showed molecular similarity values of 99% (*X. rivesi*, *X. santos*, *X. citricolum*, *X. americanum*, *X. thornei*, *X. pacificum* and *X. georgianum*) (data not shown). High inter-specific similarity values were detected in the other species—*L. wicuolea* and *L. silvestris* or *X. pseudocoxi* and *X. globosum*—which showed a similarity value of 97%. Hence, in these species, this marker could not provide clear species identification, and other sequences and integrative taxonomic approaches must be applied^28^.

The ITS1 maker showed low intra-specific molecular variability in the majority of the species studied; only some species showed a significantly low similarity (below 90%), such as *X. brasiliense* (89%), *X. inaequale* (80%), *X. chambersi* (87%), and *L. biformis* (85%). Unfortunately, because no data were available to confirm that these cases were misidentifications, further research is needed to confirm this high molecular variability. ITS sequences have been a prominent choice for species identification because this region is one of the most variable nuclear loci, and the availability of universal primers that work with most nematodes^29^ has contributed to its extensive use (Table S4). However, the high length and sequence variability between Longidoridae species complicates the construction of a plausible alignment of this region. Thus, this region appears to be better for species delimitation than for phylogenetic studies^17, 29^.

**Figure 1:**
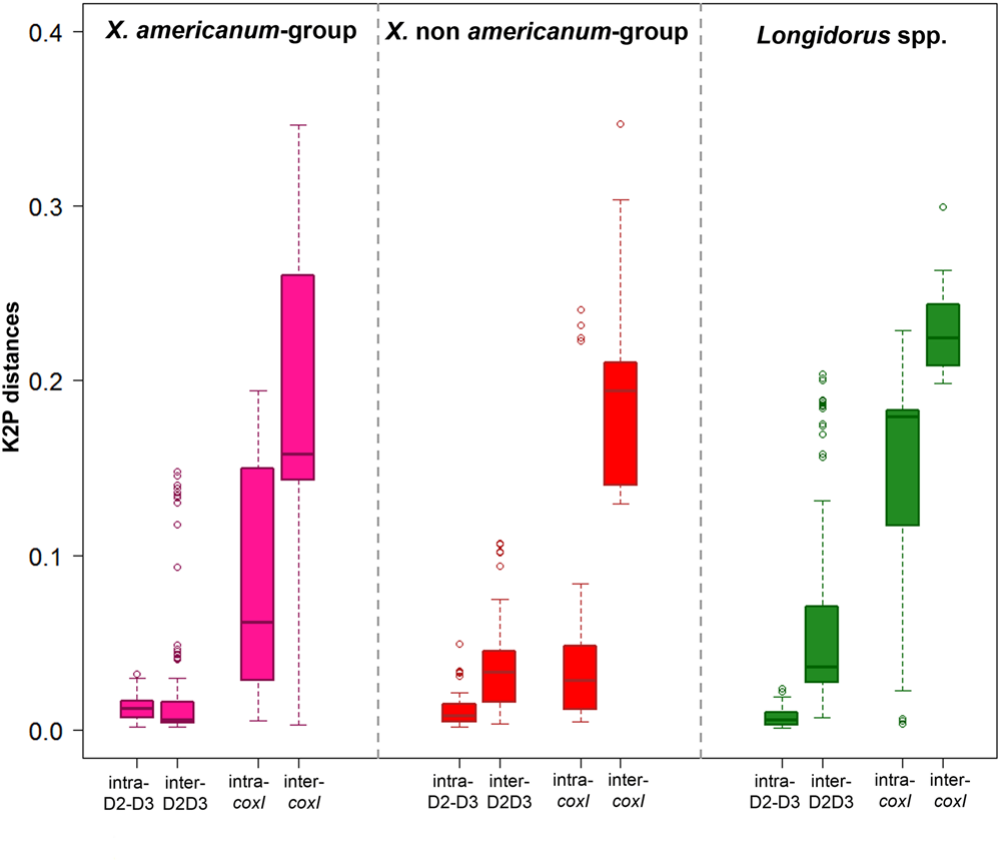
Intra- and inter-specific distance (K2P) for D2–D3 region and *coxI* markers for different groups of species within Longidoridae. Distances calculated using the biggest distance for intra-specific variability for each individual (sequence) among the sequences for the same species and the smallest distance among species for each individual. The box shows the third (Q3) and first (Q1) quartile range of the data and the median. Whiskers indicate minimum and maximum values of the data. Data falling outside the box and whiskers (circle) range are plotted but considered outliers.

Maximum intra- and minimum inter-specific distances for each *coxI* and D2–D3 sequences are shown in Fig. 1, which shows that higher molecular variability for K2P distance was associated with partial *coxI* than with D2–D3 region for intra- and inter-specific comparisons. As discussed above, the range of intra- and inter-specific distances in the *X. americanum* group was minimal for the D2–D3 region. Importantly, the difference between intra- and inter-specific distances in the *X*. non-*americanum* is large and non-overlapping. The intra-specific variability in *coxI* is largely attributable to *X. italiae* in this group.

### Barcoding

To evaluate how well various barcoding tools perform for Longidoridae, we analyzed datasets for species that had been previously identified using integrative taxonomy and in addition data for Longidoridae deposited in GenBank. Three software packages were tested: Weka, Spider and phylogenetic trees topology based on MrBayes. We included and excluded the *X. americanum* group to understand the effect of these close-related species in our analysis. Our results suggest that DNA barcoding could be a powerful tool for the majority of species in Longidoridae using several approaches: (a) supervised machine learning methods; (b) distance threshold methods and (c) monophyly for species with more than two sequences in phylogenetic trees. However, barcoding results were highly dependent on the selected molecular marker and the technique used (Tables 2 and 3). Both mitochondrial and ribosomal sequences have been used as barcoding regions for nematodes in studies with smaller sample sizes and a larger phylogenetic range^30, 31^. Since our sequences were all derived from single vouchered specimens and are of high quality because we sequenced PCR products from both ends, the present reference database could also be a valuable tool for validating field collections^32^. The marker could also be used for soil nematode metabarcoding^33, 34^. The majority of our sequences for partial *coxI* are 400 bp long, which is in the range of appropriate size suggested by iBOL data quality: length of finished sequence must be >75% of approved marker length (*e.g*., 500 bp for *coxI*), with an expectation of 2X coverage (http://ibol.org/about-us/how-ibol-works/). With this sequence, we could clearly re-identify the majority of species, except for closely related species in the *X. americanum* group or species that were probably misidentified. The D2–D3 marker showed considerable sequence similarity in the *X. americanum* group, and for this reason two datasets were studied—one with all sequences and other excluding these sequences—to check the validity for the *X*. non-*americanum-*group species (Tables 2 and 3).

**Table 2:** Accuracies (% correctly identified sequences from the test dataset) for barcoding in Longidoridae using the program Weka v.3.8.0. The datasets included all sequences of accessions that were identified to the species level and was divided into 80% as train set and 20% as test.

Dataset^1^	Jrip	J48	Naïve Bayes	Iterative Classifier Optimizer
Cytochrome oxidase 1	78.43	82.35	80.39	88.24
D2 and D3 expansion segments of the 28S	63.06	84.69	36.03	94.59
D2 and D3 expansion segments of the 28S (excluding *X. americanum*-group)	69.74	88.16	36.84	96.05

^1^ *X. brevicolle* species complex was excluded from the analysis.

**Table 3:** Accuracies for barcoding in Longidoridae using SPIDER package and tree-based comparison for monophyly using Bayesian inference.

Dataset	Number of species	Number of sequences	Near Neighbour		Best Close Match^1^				Sequences with inter-intra < = 0	Optimal differences for barcoding^2^	MrBayes phylogeny^3^
			*False*	True	Ambiguous	Correct	Incorrect	No id			
Cytochrome oxidase 1	42	253	*3*	250 (99.9%)	0	189 (74.7%)	2	62	58 (22.9%)	6.36%	92.9% (39/42)
D2 and D3 expansion segments of the 28S^4^	111	560	*24*	536 (95.7%)	18	503 (89.8%)	19	20	138 (24.7%)	2.87%	90.1% (100/111)
D2 and D3 expansion segments of the 28S (excluding *X. americanum*-group)	88	384	*11*	373 (99.9%)	7	354 (92.2%)	6	17	37 (9.6%)	2.04%	100% (88/88)

Accuracy is defined as the percentage of sequences correctly assigned to their species in the case of Near Neighbour and Best Close Match. For the tree-based method, the accuracy was expressed as the percentage of species with more than one sequence that grouped as monophyletic in their respective molecular marker tree. ^1^Threshold based criterion of 1%. ^2^Experimental script in SPIDER. ^3^Percentage of species monophyletic to the respective tree. ^4^ *X. brevicolle* species complex excluded from the analysis.

The *coxI* and D2–D3 markers performed differently depending on the barcoding techniques used. The learning methods implemented in the Weka package achieved similar results for the *coxI* marker, ranging from 78.43% to 88.24% (Table 2). The performance of classification by machine learning was not strongly influenced by the presence of *X. americanum*-group sequences (384 *vs*. 560 sequences in D2–D3) (Table 2). The Bayesian-based method naïve Bayes classifier^35^ did not perform well with the D2–D3 data including or excluding the *X. americanum* group (36.03 and 36.84% of sequences assigned to correct species). The best classifier was the iterative classifier optimizer^36^ with 94.59 to 96.05% of sequences assigned to the correct species, followed by the decision tree C4.5 (J48)^37^ and the rule-based RIPPER (Jrip)^38^.

Using the Spider package, the Near Neighbour method showed very good accuracy for *coxI*, with almost 100% of correct identifications. Best Close Match performed less well. For both methods, the exclusion of the *X. americanum* group increased accuracy (Table 3). These results showed the potential for barcoding with these software packages for the majority of our species using both markers. In the case of MrBayes, phylogenetic analysis for species with more than one sequence showed that 92.9% of our species presented a monophyletic position in the tree for *coxI*. This performance was similar for the D2–D3 marker when both including (90.1%) and excluding the *X. americanum* group in Longidoridae (100%) (Table 3).

The knowledge of intra- and inter-specific molecular variability is important to detect misidentifications or cryptic speciation in different nematodes groups. Approximately a quarter of the sequences for *coxI* and D2–D3 region including *X. americanum* group showed a larger intra-specific than inter-specific molecular diversity; while an approximately 10% of the sequences was for D2–D3 region excluding *X. americanum* group (Table 3). Even with these differences, the performance was good and probably these molecular differences included the important molecular variability of some species, low intra-specific variability in others (species from the *X. americanum* group), poorly corrected sequences from chromatograms or sequences from PCR cloning products and, in some cases, incorrect identifications deposited in GenBank. Using an experimental script provided by the R package Spider, we were able to calculate the approximate optimal molecular differences for barcoding, which were 6.36% for *coxI* and 2.87% and 2.04% for D2–D3 when including the *X. americanum* group or excluding it, respectively (Table 3). Although this script is experimental and should be used with caution, our integrative taxonomic identifications in Longidoridae support these values^9, 20, 28, 39, 40^.

### Phylogeny of Longidoridae using nuclear and mitochondrial sequence data

**Figure 2:**
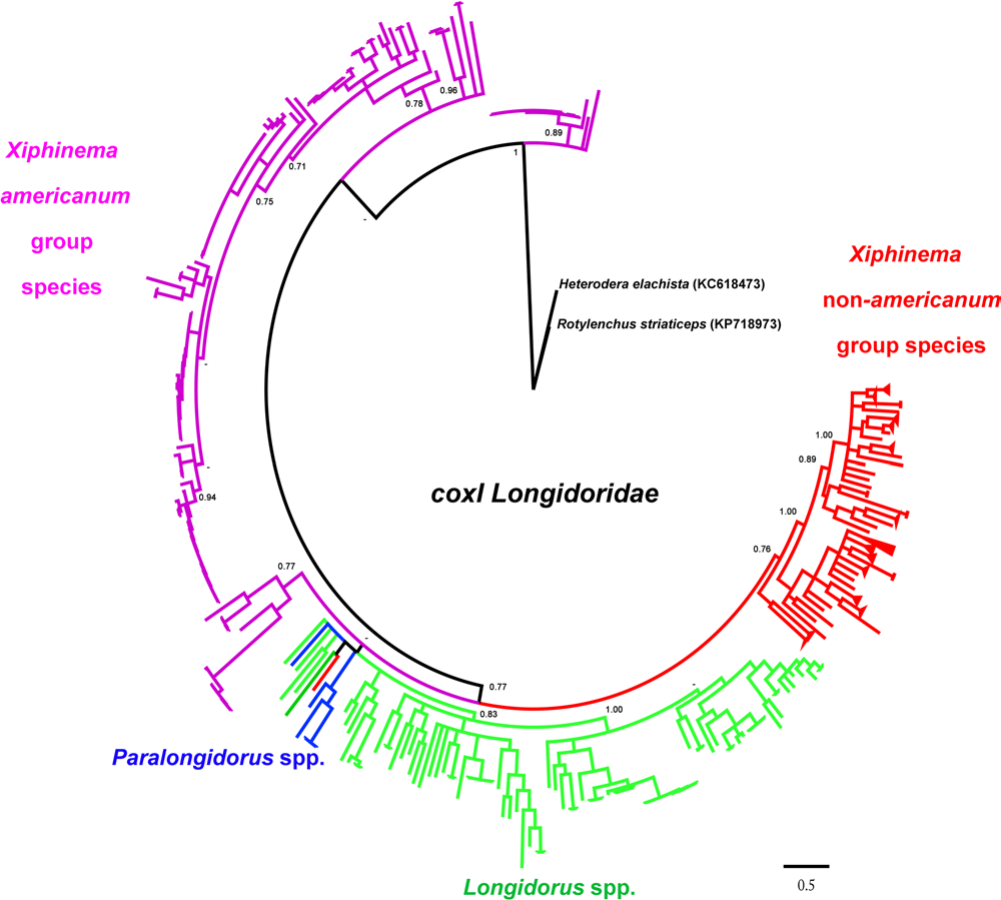
Phylogenetic relationships within Longidoridae. Bayesian 50% majority rule consensus tree as inferred from analysis of the partial *coxI* sequence alignment under a TrN + I + G model. Posterior probabilities more than 0.70 are given for appropriate clades.

The phylogeny obtained using the *coxI* fragment (583 sequences) showed a monophyletic clade for the *X*. non-*americanum*-group species and a clade for *Paralongidorus* and *Longidorus* species, while the *X. americanum* group was paraphyletic (Fig. 2). However, all clades were weakly supported (<0.95 Bayesian probability values (BPP)). The phylogenies at the species level relationship generally supported the phylogenetic relationships among groups of species in *Xiphinema* more than in *Longidorus* reported in former papers (Fig. S1)^6, 9, 11, 28, 39, 40^. Nevertheless, in this wider analysis, we could not clearly determine groupings such as *X. brevicolle* ‘complex’ (nested among *X. diffusum*, *X. taylori*, and *X. incognitum*), and some entries for *X. rivesi* (from different geographical locations) following the corrections performed by Orlando *et al*. for the *X. americanum* group (Fig. S1), as one *X. rivesi* sequence (AM086697) was considered as *X. floridae* (AM086696)^26^. In addition, *Xiphinema* sp. 5 studied by Orlando *et al*.^26^ nested inside *Longidorus*. However, when BLASTn was performed on GenBank, this sequence matched as a *Xiphinema* sp. The separation among species was remarkable, with the exception of a few species in the *X. americanum* group, using a phylogenetic approach. The base saturation (third nucleotide position in each codon) and the short fragment used in this study could be responsible for this lack of phylogenetic resolution at the genus level and between *X. americanum* and *X*. non-*americanum* group inside the genus *Xiphinema*. Additionally, different mutation rates in the mitochondrial genome and the wide evolutionary differences within these studied groups could complicate the phylogeny. A dataset excluding the third codon position did not improve the phylogeny, and in fact made it worse because of the low phylogenetic signal (Fig. S2). Probably, a possible improvement in the phylogenetic relationships among genera in Nematoda could be obtained using full mitochondrial genomes^41, 42^.

**Figure 3:**
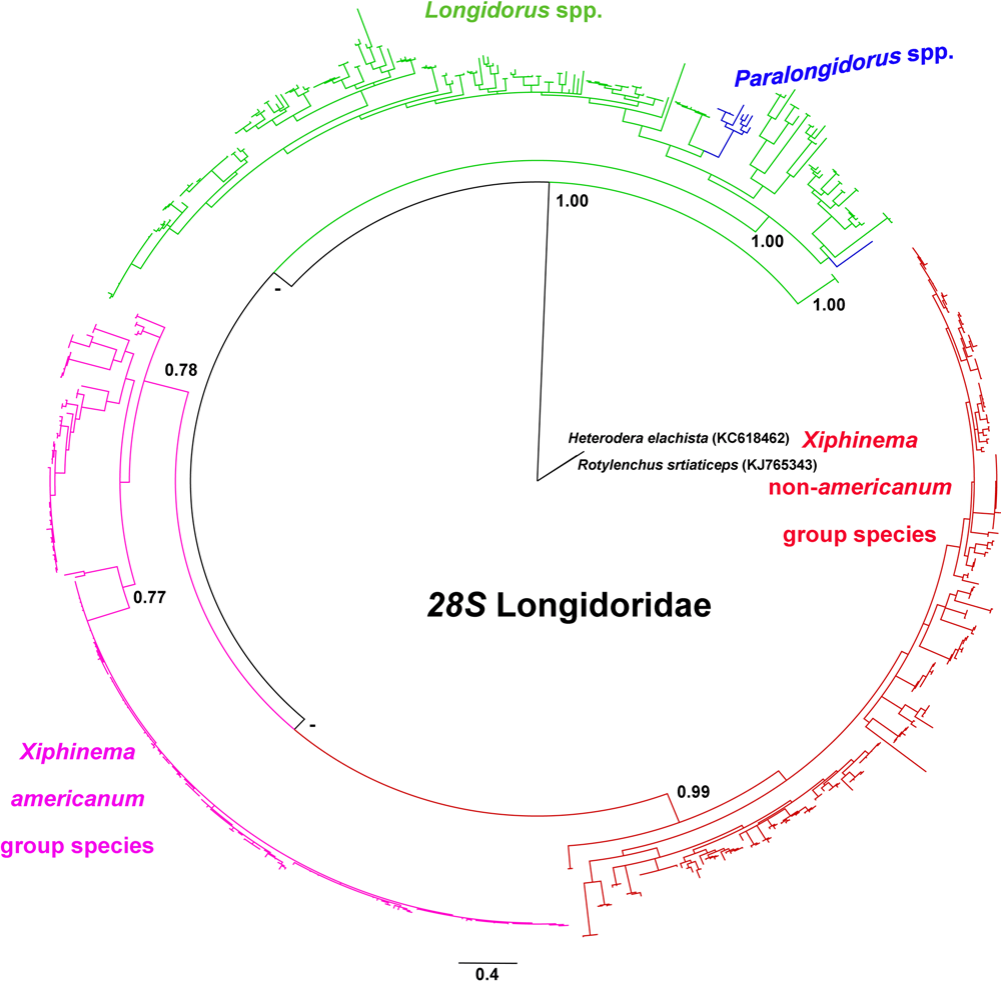
Phylogenetic relationships within Longidoridae. Bayesian 50% majority rule consensus tree as inferred from analysis of the D2–D3 region alignment under a GTR + I + G model. Posterior probabilities more than 0.70 are given for appropriate clades.

The phylogeny of nuclear ribosomal marker (D2–D3) based on 1085 sequences of Longidoridae showed a similar pattern of separation among genera (Figs 3 and S3) after corrections for some misidentified species (*X. cretense* and *X. diversicaudatum*)^43, 44^. However, here, the separation for some species was better than in the *coxI* tree, since the *X*. non-*americanum*-group species and *Longidorus-Paralongidorus* (with the exception of *L. laevicapitatus*) were clearly separated into two well-supported clades (Figs 3 and S3). However, the *X. americanum* group formed a clade that is, however, weakly supported (≤0.90 BPP). As in the analysis with *coxI*, the genus *Paralongidorus* was nested among the *Longidorus* spp. clade. *Xiphinema americanum* s. s. species formed a low supported clade (0.77) (Fig. S3). As mentioned before, this group of species showed low nucleotide variability, probably because of a short speciation time among these species. *Paralongidorus* species formed a well-supported clade (1.00 BPP) inside *Longidorus*, with the exception of *P. bikanerensis*. This phylogeny is similar to others for Longidoridae^9, 39, 45–47^. Longer sequences probably need to be added in order to address this problem of deep resolution, but major clades have been clearly resolved using a more slowly evolving gene such as 18S. Recently, the sequencing of four additional mitogenomes of Longidoridae supported a similar phylogenetic pattern of *Paralongidorus* being most closely related to *Longidorus*, both associated with the *Xiphinema* species^48^.

## Conclusions

This is the first broad study of the variability of molecular markers used for phylogenetic relationships and the identification of Longidoridae. This research significantly increases the number of *coxI* sequences available for Longidoridae using integrative taxonomic approaches with voucher specimens and the combination of several unequivocal molecular markers (*coxI*, D2–D3 region and ITS1, in some cases) from one individual nematode. The ITS1 region showed promise for barcoding and species identification because of the clear molecular variability among species. However, difficulties with obtaining an unequivocal alignment limit its usefulness beyond BLASTn-like searches. In addition, we revealed problems for species delimitation in Longidoridae, as well as phylogenetic relationships using *coxI* and D2–D3 regions. However, in shallow phylogenetic relationships (close to the external branches of the tree) or for a restricted number of species, these markers gave good results. Several barcoding methods showed the utility of *coxI* and D2–D3 for species identification, except for some species in the *X. americanum* group (for which more studies are necessary for longer sequences or different markers). Our results suggest that the use of more than one molecular marker is essential for the correct identification of Longidoridae unless integrative taxonomical approaches are employed.

## Material and Methods

### Samples and nematode extraction

Nematode soil samples were collected from 2007 to 2016, mainly in Spain but also in Greece, Japan, the USA, Russia and Italy, from the rhizosphere of a wide variety of plants, including both agriculture and natural ecosystems (Tables 1 and S1). At each site, several subsampling points were randomly selected for soil sampling in an area of 5 m^2^. Soil samples were collected with a shovel discarding the upper 5-cm top soil profile from a 5- to 40-cm depth, in the close vicinity of active roots. To obtain a representative soil sample per site, all subsample soils were thoroughly mixed before nematode extraction. Nematodes from the soil were extracted from a 500-cm^3^ sub-sample using the magnesium sulphate centrifugal-flotation method^49^. The extracted nematodes were identified by selecting adult nematode specimens belonging to Longidoridae. Nematodes were fixed in 4% formaldehyde, processed with glycerin^50^, and identified by morphological traits to the genus or species level. Some additional nematodes from the same morphotype were not fixed and were used for molecular studies from each site.

### DNA extraction and PCR conditions

For molecular analyses, to avoid complications from mixed species populations in the same sample, at least two live nematodes from each sample were temporarily mounted on a drop of 1 M NaCl containing glass beads (to avoid crushing the nematode). Here, diagnostic morphological characteristics were observed and measurements were taken to confirm species identity. The slides were dismantled and DNA was extracted. Nematode DNA was extracted from single individuals and PCR assays were conducted as described by Castillo *et al*.^51^. The portion of the partial *coxI* gene was amplified, as described by Lazarova *et al*.^21^ using the primers COIF (5′-GATTTTTTGGKCATCCWGARG-3′), COIR (5′-CWACATAATAAGTATCATG-3′), XIPHR1 (5′-ACAATTCCAGTTAATCCTCCTACC-3′) or XIPHR2 (5′-GTACATAATGAAAATGTGCCAC-3′) and as Bowles *et al*.^23^ using primers JB3 (5′-TTTTTTGGGCATCCTGAGGTTTAT-3′) and JB4 (5′-TAAAGAAAGAACATAATGAAAATG-3′). PCR cycle conditions for mtDNA were as described by Lazarova *et al*.: 1 cycle of 94 °C for 1 min, 50 °C for a further 1 min and 72 °C for 2 min. This was followed by 40 cycles of 94 °C for 1 min, 45 °C for 1 min and 72 °C for 2 min. The PCR was completed with a final extension phase of 94 °C for 1 min, 45 °C for 1 min and 72 °C for 5 min^21^. The D2–D3 region was obtained using a protocol and primers described in Archidona-Yuste *et al*.^9, 39^. PCR products were purified after amplification using ExoSAP-IT (Affmetrix, USB products) and used for direct sequencing in both directions. The resulting products were run on a DNA multicapillary sequencer (Model 3130XL genetic analyser; Applied Biosystems, Foster City, CA, USA), using the BigDye Terminator Sequencing Kit v.3.1 (Applied Biosystems, Foster City, CA, USA), at the Stab Vida sequencing facilities (Caparica, Portugal). The newly obtained sequences were submitted to the GenBank database under accession numbers indicated on the phylogenetic trees and Tables 1 and S1.

### Nucleotide variability analyses

A total of 577, 257, and 261 sequences from 112, 65 and 44 species of Longidoridae were used to calculate the intra- and inter-specific molecular variability of 28S, ITS1 and *coxI*, respectively. For intra-specific molecular variability, one dataset from each species with more than one available sequence (Tables S2–S4) was created and aligned using MAFFT v. 7.2^52^ with default parameters. Then, pairwise divergence among taxa were computed as a percentage of sequence similarity, singletons sites and parsimony informative sites using the program MEGA v. 7.0^53^ (Tables S2–S4). Additionally, for *coxI*, *p*-distance was calculated for each codon position. For inter-specific molecular variability, four datasets were created, including sequences from the *X*. non-*americanum* group, *X. americanum* group, *Longidorus* spp. and *Paralongidorus* spp. Nucleotide variability indices were calculated in the same way as the intra-specific molecular variability after grouping the different species in each dataset (MEGA v.7.0). “Spider” package^54^ with R version 3.1.1 freeware (R Core Development Team; CRAN, http://cran.r-project.org)^55^ generates two statistics for each sequence (individual) in the dataset: the furthest intra-specific distance among its own species and the closest, non-conspecific (i.e., inter-specific distance). These data were used to create Fig. 1 among makers and species groups.

### Barcoding analyses

Species without clear taxonomic status (*X. brevicolle*) and sequences considered misidentifications using several phylogenetic analyses^9, 26, 39, 43, 44^, as well as sequences with less than 300 bp in the D2–D3 fragment, were excluded from the analysis. Two datasets were used, corresponding to the *coxI* and D2–D3 regions. Several barcoding methods were used to test the utility of these molecular markers for species identification: (*i*) supervised machine learning methods to classify species following the method explained by Weitschek *et al*.^56^ using the Weka machine learning software^55^, which includes a collection of supervised classification methods. Jrip, J48, and naïve Bayes were used as supervised classification methods. The dataset included all species identified with all molecular variability using a test option for the dataset with a percentage split of 80% train set of sequences and 20% as test sequences, this option is allowed in Weka v.3.8.0^57^ using the following Weka classifiers: (1) the rule-based RIPPER (Jrip)^38^; (2) the decision tree C4.5 (J48)^37^; (3) the Iterative Classifier Optimizer^57^; and (4) the Bayesian-based method naïve Bayes^35^. (*ii)* Tests of barcoding “best close match”^58^, nearest-neighbour identification^59^, and a standard threshold cut-off for species separation was determined using the function “localMinima” (this function determines possible thresholds from the distance matrix for an alignment) using a dataset for both the *coxI* and D2–D3 regions (including and excluding the *X. americanum* group) using the indications and principal functions implemented in the “spider” package^54^ with R version 3.1.1 freeware (R Core Development Team; CRAN, http://cran.r-project.org)^55^. Additionally, *iii)* phylogenetic trees conducted using MrBayes were analysed for species monophyly and species congruence for species with more than one available sequence. For this analysis, species not forming a monophyletic clade were considered not well identified, and the number of divergent sequences was annotated.

ITS1 sequences were excluded from all analyses because of the high divergence degree and difficulties with regard to phylogenies and correct alignments. However, a molecular variability table was considered in order to elucidate the molecular diversity of this marker in Longidoridae.

### Phylogenetics analyses

Nucleotide data sets consisted of the partial *coxI* fragments for barcoding species in Longidoridae and of protein coding fragments. Outgroup taxa were *Heterodera elachista* and *Rotylenchus striaticeps*. The newly obtained and published sequences for each gene were aligned using MAFFT v. 7.2^52^ with default parameters. Sequence alignments were manually edited using BioEdit^57^. Phylogenetic analyses of the sequence data sets were performed based on Bayesian inference (BI) using MrBayes 3.1.2^60^. The best fitting model of DNA evolution was obtained using jModelTest v. 2.1.7^61^ with the Akaike Information Criterion (AIC). The Akaike-supported model, the base frequency, the proportion of invariable sites, and the gamma distribution shape parameters and substitution rates in the AIC were then used in phylogenetic analyses. BI analysis under a Tamura-Nei with a proportion of invariable sites and a gamma-shaped distribution (TrN + I + G) model for *coxI* mtDNA was run for 4 × 10^6^ generations, while for the first and second nucleotide for each codon a transversion model with a proportion of invariable sites and a gamma-shaped distribution (TVM + I + G) was used, with 10 × 10^6^ generations. The general time reversible model with a proportion of invariable sites and a gamma-shaped distribution (GTR + I + G) using 10 × 10^6^ generations was used for the D2–D3 maker. The Markov chains were sampled at intervals of 100 generations. Two runs were performed for each analysis. After discarding burn-in samples and evaluating convergence, the remaining samples were retained for further analyses. The topologies were used to generate a 50% majority rule consensus tree. Posterior probabilities (PP) are given in appropriate clades. Trees were visualized using TreeView^62^ and FigTree v1.4.2 (http://tree.bio.ed.ac.uk/software/figtree/).

## Electronic supplementary material

Supporting Information
