# Supplementary material for: The utility of mtDNA and rDNA for barcoding and phylogeny of plant-parasitic nematodes from Longidoridae (Nematoda, Enoplea)
Source: Sci Rep. 2017 Sep 7;7:10905. doi: 10.1038/s41598-017-11085-4 (PMC5589882; doi:10.1038/s41598-017-11085-4)
Supplement: Supplementary file 1 — Supporting Information [file 41598_2017_11085_MOESM1_ESM.pdf]

<u>Supporting information</u>

The utility of mtDNA and rDNA for barcoding and phylogeny of plant-parasitic nematodes from Longidoridae (Nematoda, Enoplea)

Palomares-Rius, J.E.[1], Cantalapiedra-Navarrete, C.[1], Archidona-Yuste, A.[1], Subbotin, S.A.[2,3] and Castillo, P.[1]

[1]Instituto de Agricultura Sostenible (IAS), Agencia Estatal Consejo Superior de Investigaciones Científicas (CSIC), Avda. Menéndez Pidal s/n, 14004, Córdoba, Spain.

[2]Plant Pest Diagnostic Center, California Department of Food and Agriculture, 3294 Meadowview Road, Sacramento, CA 95832-1448, USA

[3]Center of Parasitology of A.N. Severtsov Institute of Ecology and Evolution of the Russian Academy of Sciences, Leninskii Prospect 33, Moscow, 117071, Russia

Table S1. Taxa studied for dagger and needle nematodes species of the family Longidoridae and sequences of cytochrome *c* oxidase subunit 1 (*coxI*) used in this study. For new sequences, species identifications were based on morphology and barcoding using D2-D3 expansion segments of 28S rDNA[1].

Nematode species	Sample code	Locality	Host plant	GenBank accession numbers 28S	coxI
Genus ***Xiphinema*** spp.					
1. ***Xiphinema adenohystherum***	SORIA	Arévalo de la Sierra, Soria province, Spain	european holly	KC567164	KY816588
Xiphinema adenohystherum	ALMAG	Almagro, Ciudad Real province, Spain	wild olive	*[2]	KY816589
Xiphinema adenohystherum	AR086	Prado del Rey, Cádiz province, Spain	wild olive	*	KY816590
Xiphinema adenohystherum	AR078	Almodóvar, Córdoba province, Spain	wild olive	*	KY816591
Xiphinema adenohystherum	IASNB	Jerez de la Frontera, Cádiz province, Spain	wild olive	*	KY816592
2. ***Xiphinema americanum***	-	USA	orchard fruit	-	AM086693
Xiphinema americanum	CD48	California, USA	plum	KX263169	KX263063
Xiphinema americanum	CD82b	California, USA	grape	KX263145	KX263064
Xiphinema americanum	CD100	California, USA	grape	KX263151	KX263062
Xiphinema americanum	CD1074	Georgia, USA	-	KX263168	KX263050
Xiphinema americanum	CD1078	Texas, USA	-	KX263173	KX263054
Xiphinema americanum	CD1244	California, USA	orange	KX263171	KX263053
Xiphinema americanum	XA34	California, USA	walnut	KX263172	KX263059
Xiphinema americanum	XA70	California, USA	cherry	KX263146	KX263047
Xiphinema americanum	CD1816a	Stavropol Krai, Kislovodsk, Russia	cherry	KX263179	KX263106
Xiphinema americanum	CD1816b	Stavropol Krai, Kislovodsk, Russia	cherry	KX263179	KX263107
3. ***Xiphinema andalusiense***	ARO93	Belmez ,Córdoba, Spain	wild olive	KX244884	KY816593
Xiphinema andalusiense	00419	Andújar, Jaén, Spain	wild olive	KX244885	KY816594
Xiphinema andalusiense	AR108	Villaviciosa ,Córdoba, Spain	wild olive	KX244888	KY816595
4. ***Xiphinema astaregiense***	J0174	Jerez de la Frontera, Cádiz province, Spain	sunflower	KP268955	KP268977
5. ***Xiphinema baetica***	LOMAS	Hinojos, Huelva province, Spain	stone pine	KC567165	KY816596
Xiphinema baetica	HATRA	Villamanrique de la Condesa, Huelva, Spain	cork oak	KC567166	KY816597
6. ***Xiphinema bakeri***	CD852	California, USA	-	KF292277	KF292305
Xiphinema bakeri	CD840	California, USA	*Salix* sp.	KF292278	KF292306
7. ***Xiphinema belmontense***	MOUCH	Merza, Pontevedra province, Spain	chestnut	KC567171	KY816598
8. ***Xiphinema brevicolle***	-	Brazil	*Coffeae arabica*	-[3]	AM086707
Xiphinema brevicolle	-	Breclav, Czech Republic	grapevine	HM163209	HM163206
Xiphinema brevicolle	CD1816	Stavropol Krai, Kislovodsk, Russia	cherry	KX263179	KX263106
Xiphinema brevicolle	CD1816	Stavropol Krai, Kislovodsk, Russia	cherry	KX263179	KX263107
Xiphinema brevicolle	-	Chiba, Yachimata, Japan	*Ilex crenata*	-	AB675670
Xiphinema brevicolle	-	Chiba, Yachimata, Japan	*Ilex crenata*	-	AB675672
Xiphinema brevicolle	-	Chiba, Yachimata, Japan	*Ilex crenata*	-	AB675673

Nematode species	Sample code	Locality	Host plant	GenBank accession numbers	
Xiphinema brevicolle	-	Chiba, Sosa, Japan	*Ilex crenata*	-	AB604337
Xiphinema brevicolle	-	Chiba, Togane, Japan	*Ilex crenata*	-	AB655671
Xiphinema brevicolle	-	Chiba, Yachimata, Japan	*Ilex crenata*	-	AB675669
9. ***Xiphinema brevisicum***	CARIX	Merza, Pontevedra province, Spain	oak	KP268962	KP268979
10. ***Xiphinema browni***	-	Moca, Slovakia	grapevine	-	KU250158
Xiphinema browni	-	Slovakia	grapevine	-	AM086708
Xiphinema browni	NSB1	Kurdejov, Czech Republic	grapevine	KU250151	GU222424
11. ***Xiphinema cadavalense***	ST077	Espiel, Córdoba province, Spain	cultivated olive	KX244932	KY816599
12. ***Xiphinema californicum***	CD3	California, USA	cherry	KX263143	KX263071
Xiphinema californicum	CD231	California, USA	lemon	KX263166	KX263067
Xiphinema californicum	CD1247	California, USA	orange	KX263156	KX263073
Xiphinema californicum	CD1818	California, USA	*Acer* sp.	KX263153	KX263069
Xiphinema californicum	XA4	California, USA	peach	KX263165	KX263077
Xiphinema californicum	XA5	California, USA	plum	KX263162	KX263078
Xiphinema californicum	XA8	California, USA	plum	KX263164	KX263079
Xiphinema californicum	XA42	California, USA	walnut	KX263159	KX263081
Xiphinema californicum	XA44	California, USA	cherry	-	KX263080
13. ***Xiphinema celtiense***	AR083	Adamuz, Córdoba province, Spain	wild olive	KX244889	KY816600
Xiphinema celtiense	AR082	Adamuz, Córdoba province, Spain	wild olive	KX244890	KY816601
14. ***Xiphinema* cf. *americanum***	-	Florida, USA	-	-	AM086689
Xiphinema* cf. *americanum	-	South Africa	-	-	AM086690
Xiphinema* cf. *americanum	-	Florida, USA	-	-	AM086691
Xiphinema* cf. *americanum	-	Nepal	-	-	AM086704
15. ***Xiphinema chambersi***	-	Jekyll Island, Georgia USA	*Quercus virginiana*	-	KU764412
Xiphinema chambersi	-	Jekyll Island, Georgia USA	*Quercus virginiana*	-	KU764413
Xiphinema chambersi	-	Jekyll Island, Georgia USA	*Quercus virginiana*	-	KU764414
Xiphinema chambersi	-	Jekyll Island, Georgia USA	*Quercus virginiana*	-	KU764415
Xiphinema chambersi	-	Jekyll Island, Georgia USA	*Quercus virginiana*	-	KU764416
Xiphinema chambersi	-	Jekyll Island, Georgia USA	*Quercus virginiana*	-	KU764417
Xiphinema chambersi	-	Jekyll Island, Georgia USA	*Quercus virginiana*	-	KU764418
Xiphinema chambersi	-	Jekyll Island, Georgia USA	*Quercus virginiana*	-	KU764419
16. ***Xiphinema cohni***	J0126	Puerto de Sta. María, Cádiz province, Spain	grapevine	KC567173	KY816602
17. ***Xiphinema conurum***	ST45V	Sorbas, Almería province, Spain	cultivated olive	KX244892	KY816603
18. ***Xiphinema costaricense***	ACC86	Guayabo. Turrialba. Cartago	forest	KX931056	KY816604
Xiphinema costaricense	ACC46	Santa Rosa. Limón. Limón	cocoa	KX931057	KY816605
19. ***Xiphinema coxi europaeum***	AR020	Hinojos, Huelva province, Spain	wild olive	KC567174	KY816606
Xiphinema coxi europaeum	H0027	Almonte, Huelva province, Spain	cork oak	KC567177	KY816607
20. ***Xiphinema cretense***	AR039	Hersonisos province	wild olive	KJ802878	KY816608
21. ***Xiphinema dentatum***	-	Silnicna, Czech Republic	Forest soil	EU781538	EU781537

Nematode species	Sample code	Locality	Host plant	GenBank accession numbers	
22. ***Xiphinema diffusum***	-	Australia	Grapevine	-	AM086700
Xiphinema diffusum	-	Brazil	Orchard fruit	-	AM086699
Xiphinema diffusum	-	China	Unknown	-	AM086701
Xiphinema diffusum	-	Brazil	Unknown	-	AM086698
23. ***Xiphinema diversicaudatum***	-	Bile Podoli, Czech Republic	sour cherry	-	GU222421
Xiphinema diversicaudatum	CD1311	Varenikovskaya, Russia	*Salix alba*	-	KF292294
Xiphinema diversicaudatum	CD1308	Saratov, Russia	*Acer negundo* L.	KF292279	KF292298
Xiphinema diversicaudatum	CD1283	Moscow region, Serpukhov, Russia	*Salix alba* L.	KF292280	KF292297
Xiphinema diversicaudatum	CD1335	Moscow region, Serpukhov, Russia	*Populus nigra L.*	-	KF292296
Xiphinema diversicaudatum	CD1330	Moscow region, Klyazma, Russia	*Ulmus glabra* Huds	-	KF292301
Xiphinema diversicaudatum	CD1331	Voronezh region, Ramon, Russia	*S. alba*	-	KF292295
Xiphinema diversicaudatum	CD1309	Voronezh region, Ramon, Russia	*Alnus glutinosa* L.	-	KF292302
Xiphinema diversicaudatum	CD1338	Krasnodar Territory, Sochi, Russia	*Citrus unshiu* Marc.	-	KF292287
Xiphinema diversicaudatum	CD1310	Krasnodar Territory, Sochi, Russia	*Medicago lupulina* L.	-	KF292292
Xiphinema diversicaudatum	CD1332	Krasnodar Territory, Sochi, Russia	*Urtica dioica* L.	-	KF292286
Xiphinema diversicaudatum	CD1312	Krasnodar Territory, Sukko, Russia	*Sambucus nigra* L.	-	KF292284
Xiphinema diversicaudatum	CD1313	Krasnodar Territory, Sukko, Russia	*Corylus avellana* L.	-	KF292290
Xiphinema diversicaudatum	CD1336	Krasnodar Territory, Sukko, Russia	*P. alba*	-	KF292288
Xiphinema diversicaudatum	CD1348	Predela, Bulgaria	Meadow plants	-	KF292303
Xiphinema diversicaudatum	CD1349	Bachevo, Perivol, Bulgaria	*Picea abies* L.	-	KF292304
24. ***Xiphinema duriense***	ST02C	Gibraleón, Huelva province, Spain	cultivated olive	KP268963	KY816609
25. ***Xiphinema floridae***	-	USA	Orchard fruit	-	AM086696
Xiphinema floridae	-	USA	Orchard fruit	-	AM086697
26. ***Xiphinema georgianum***	-	USA	Orchard fruit	-	AM086695
27. ***Xiphinema gersoni***	H0059	Almonte, Huelva province, Spain	eucalyptus	KC567180	KY816610
28. ***Xiphinema herakliense***	OLEA8	Vathy Rema, Crete Island , Greece	wild olive	KM586345	KY816611
Xiphinema herakliense	OLEA17	Agiofarago, Crete Island , Greece	wild olive	KM58636	KY816612
Xiphinema herakliense	OLE18	Agiofarago, Crete Island , Greece	wild olive	KM58639	KY816613
29. ***Xiphinema hispanum***	00419	Andújar, Jaén province, Spain	wild olive	GU725074	KY816614
30. ***Xiphinema hispidum***	AR098	Bollullos par del Condado, Huelva province, Spain	grapevine	KC567181	KY816615
Xiphinema hispidum	H0026	Rociana del Condado, Huelva province, Spain	grapevine	HM921366	KY816616
31. ***Xiphinema inaequale***	-	Baijnath. India	*Terminalia bellirica*	-	HM163207
32. ***Xiphinema incertum***	-	Jerez de la Frontera, Cádiz province, Spain	-	-	JQ990058
33. ***Xiphinema incognitum***	-	China	litchi	-	AM086705
Xiphinema incognitum	-	China	litchi	-	AM086706
34. ***Xiphinema index***	IN393	Moriles, Córdoba province, Spain	grapevine	HM921398	HM921380
Xiphinema index	IN116	Montemayor, Córdoba province, Spain	grapevine	HM921399	HM921381
Xiphinema index	IN116	Montemayor, Córdoba province, Spain	grapevine	HM921400	HM921382
Xiphinema index	INAUL	Aulaga, Córdoba province, Spain	grapevine	HM921401	HM921383

Nematode species	Sample code	Locality	Host plant	GenBank accession numbers	
Xiphinema index	IN499	Jerez de la Fra., Cádiz province, Spain	grapevine	HM921402	HM921384
Xiphinema index	IN474	Rociana, Huelva province, Spain	grapevine	HM921403	HM921385
Xiphinema index	INNR4	Parteolla, Sardinia province, Italy	grapevine	HM921404	HM921386
Xiphinema index	INVRM	Trexenta, Sardinia province, Italy	grapevine	HM921405	HM921387
Xiphinema index	INVRM	Trexenta, Sardinia province, Italy	grapevine	HM921406	HM921388
35. ***Xiphinema insigne***	MIYA1	Miyazaki, Japan	*Prunus* sp.	*	KY816617
36. ***Xiphinema israeliae***	AR013	Roufas province, Greece	wild olive	KJ802883	KY816618
37. ***Xiphinema italiae***	AR041	Las Tres Villas,Almería province, Spain	wild olive	KX244911	KY816619
Xiphinema italiae	AR091	Puerto Real, Cádiz province, Spain	wild olive	KX244912	KY816620
Xiphinema italiae	TUNIS	Sbitla, Kasserine, Tunisia	cultivated olive	KX062674	KY816621
Xiphinema italiae	TUN11	Sbiba, Kasserine, Tunisia	cultivated olive	KX062677	KY816622
Xiphinema italiae	APUL	Bari, Bari province, Italy	grapevine	*	KY816623
Xiphinema italiae	-	Moca, Slovakia	grapevine	FJ713153	FJ713151
38. ***Xiphinema iznajarense***	JAO25	Iznájar , Córdoba province, Spain	cultivated olive	KX244892	KY816624
39. ***Xiphinema krugi***	ACC47	Sucre, Ciudad Quesada, Alajuela	Robust star-grass	KX931061	KY816625
Xiphinema krugi	ACC13	Santa Gertrudis. Grecia. Alajuela	Sugar-cane	KX931060	KY816626
40. ***Xiphinema lambertii***	-	Baijnath, India	*Terminalia bellirica*	-	HM163208
41. ***Xiphinema luci***	IAGRQ	Benacazón, Sevilla province, Spain	rose	KP268965	KY816627
42. ***Xiphinema lupini***	H0050	Hinojos, Huelva province, Spain	grapevine	KC567183	KY816628
Xiphinema lupini	388GD	Bollullos par del Condado, Huelva province, Spain	grapevine	HM921352	KY816629
Xiphinema lupini	388GD	Bollullos par del Condado, Huelva province, Spain	grapevine	*	KY816630
43. ***Xiphinema macroacanthum***	ITAL	Brindisi, Italy	cultivated olive	*	KY816631
44. ***Xiphinema macrodora***	AR097	Santa Mª de Trassierra, Córdoba, Spain	wild olive	KU171044	KY816632
45. ***Xiphinema madeirense***	AR031	Tarifa, Cádiz province, Spain	wild olive	KP268966	KP268978
46. ***Xiphinema mengibarense***	O3C04	Mengíbar, Jaen province, Spain	Cultivated olive	KX2448893	KY816633
Xiphinema mengibarense	O30V5	Mengíbar, Jaen province, Spain	Cultivated olive	KX2448894	KY816634
47. ***Xiphinema meridianum***	11R16	Sbitla, Kasserine, Tunisia	Cultivated olive	KX062678	KY816635
48. ***Xiphinema nuragicum***	ST012	Espejo, Córdoba province, Spain	grapevine	*	KY816636
Xiphinema nuragicum	AR054	Medina Sidonia, Cádiz province, Spain	wild olive	*	KY816637
Xiphinema nuragicum	ST106	La Puebla de los Infantes, Sevilla province, Spain	cultivated olive	*	KY816638
Xiphinema nuragicum	JAO28	Antequera, Málaga province, Spain	cultivated olive	*	KY816639
Xiphinema nuragicum	AR113	Alcolea, Córdoba province, Spain	wild olive	*	KY816640
49. ***Xiphinema opisthohysterum***	AR031	Tarifa, Cádiz province, Spain	wild olive	KP268967	KY816641
Xiphinema opisthohysterum	00418	Andújar, Jaén province, Spain	grasses	JQ990040	KY816642
Xiphinema opisthohysterum	-	Almonte, Huelva province, Spain	grapevine	JQ990032	JQ990053
Xiphinema opisthohysterum	-	Andújar, Jaen province, Spain	grasses	JQ990040	JQ990054
50. ***Xiphinema pachtaicum***	PAJ40	Jerez de la Frontera, Cádiz province, Spain	grapevine	JQ990033	JQ990057
Xiphinema pachtaicum	PAIAS	Córdoba, Córdoba province, Spain.	grapevine	HM921389	HM921369
Xiphinema pachtaicum	PA393	Moriles, Córdoba province, Spain.	grapevine	HM921390	HM921370

Nematode species	Sample code	Locality	Host plant	GenBank accession numbers	
Xiphinema pachtaicum	PA393	Moriles, Córdoba province, Spain.	grapevine	HM921391	HM921371
Xiphinema pachtaicum	PAFRI	Frigiliana, Málaga province, Spain.	grapevine	HM921392	HM921372
Xiphinema pachtaicum	PA401	Jerez de la Fra., Cádiz province, Spain	grapevine	*	HM921373
Xiphinema pachtaicum	PA401	Jerez de la Fra., Cádiz province, Spain	grapevine	HM921393	HM921374
Xiphinema pachtaicum	PA500	Jerez de la Fra., Cádiz province, Spain	grapevine	HM921394	HM921375
Xiphinema pachtaicum	PA506	Trebujena, Cádiz province, Spain	grapevine	HM921395	HM921376
Xiphinema pachtaicum	PA484	Bollullos par del Condado, Huelva province, Spain	grapevine	HM921396	HM921378
Xiphinema pachtaicum	PARIO	La Guardia, Logroño province, Spain	grapevine	HM921397	HM921379
Xiphinema pachtaicum	V1	Palermo, Sicily, Italy	grapevine	KX263184	KX263108
Xiphinema pachtaicum	CD230	California, USA	lemon	KX263181	KX263109
Xiphinema pachtaicum	XA69	California, USA	plum	KX263183	KX263110
Xiphinema pachtaicum	CD49	California, USA	plum	KX263182	KX263111
Xiphinema pachtaicum	V2	Palermo, Sicily, Italy	olive	-	KX263112
Xiphinema pachtaicum	V3	Palermo, Sicily, Italy	pomegranate	-	KX263115
51. ***Xiphinema parabrevicolle***	XBRIN-	Pantanagianni, Italy	Lentisc and marram grass	JQ990042	JQ990059
52. ***Xiphinema parapachydermum***	H0018	Hinojos, Huelva province, Spain	grapevine	JQ990036	JQ990056
53. ***Xiphinema parasimile***	-	Vinogradets, Bulgaria	grapevine	-	KU250159
54. ***Xiphinema peruvianum***	-	Brazil	Coffeae Arabica	-	AM086712
Xiphinema peruvianum	-	Georgia, USA	Orchard fruit	-	AM086692
55. ***Xiphinema pseudocoxi***	AR095	Alcaracejos, Córdoba province, Spain	wild olive	KX244915	KY816643
56. ***Xiphinema pyrenaicum***	ESMEN	Cahors, Quercy province, France	grapevine	GU725073	KY816644
57. ***Xiphinema rivesi***	CASLO	Castillo de Locubín, Jaén province, Spain	cherry tree	JQ990037	KY816645
Xiphinema rivesi	00518	Moriles, Córdoba province, Spain	grapevine	HM921357	KY816646
Xiphinema rivesi	ALGUA	Alcala de Guadaira, Sevilla Province, Spain	grapevine	JQ990039	JQ990060
Xiphinema rivesi	CD1165	Minnesota, Brooklyn Park, USA	grasses and shrubs	KX263176	KX263100
Xiphinema rivesi	CD1715	California, USA	-	KX263140	KX263104
Xiphinema rivesi					KX263102
Xiphinema rivesi					KX263103
58. ***Xiphinema robbinsi***	12R28	Sbitla, Kasserine, Tunisia	cultivated olive	KX062683	KY816647
59. ***Xiphinema santos***	H0136	Rociana del condado, Huelva province, Spain	grapevine	JQ990030	JQ990055
60. ***Xiphinema setariae***	ACC09	Pueblo Nuevo de Duacarí. Guácimo. Limón	banana	KX931066	KY816648
Xiphinema setariae	CD208	Florida, USA	-	-	KX263044
61. ***Xiphinema simile***	-	Portugal	-	-	AM086709
Xiphinema simile	-	Slovakia	*Juglans regia*	-	AM086710
Xiphinema simile	-	Cejkovice, Czech Republic	cherry	-	GU222425
Xiphinema simile	-	Detkovice, Czech Republic	cherry	-	GU222426
Xiphinema simile	-	Velke Pavlovice Czech Republic	apricot	-	GU222427
Xiphinema simile	-	Serbia	Unknown	-	AM086711
Xiphinema simile	-	Velke Pavlovice, Czech republic	apricot	-	GU222427

Nematode species	Sample code	Locality	Host plant	GenBank accession numbers	
62. ***Xiphinema sphaerocephalum***	AR063	Coto Ríos, Jaén province, Spain	wild olive	*	KY816649
63. ***Xiphinema taylori***	-	Slovakia	*J. regia*	-	AM086702
Xiphinema taylori	-	Slovakia	*J. regia*	-	AM086703
64. ***Xiphinema turcicum***	ST149	San José del Valle, Cádiz province, Spain	wild olive	*	KY816650
65. ***Xiphinema turdetanense***	AR0015	Sanlúcar de Barrameda, Cádiz province, Spain	wild olive	KC567186	KY816651
66. ***Xiphinema vallense***	AR0027	Bolonia, Cádiz province, Spain	wild olive	KP268960	KY816652
Xiphinema vallense	H00003	Hinojos, Huelva province, Spain	cultivated olive	KP268961	KY816653
67. ***Xiphinema vuittenezi***	-	Slany, Czech Republic	Apple tree	EF614265	EF614265
68. ***Xiphinema*** sp.	P0011	Sbitla, Kasserine, Tunisia	Cultivated olive	KX062686	KY816654
69. ***Xiphinema sp.*** JPNH509	-	Chiba, Japan	Ilex crenata	-	AB604339
Xiphinema sp. JPNH509	-	Chiba, Japan	Ilex crenata	-	AB604338
70. ***Xiphinema* sp1** SAS2016	CD44	California, USA	plum	KX263132	KX263093
***Xiphinema* sp1** SAS2016	CD50	California, USA	plum	KX263135	KX263086
***Xiphinema* sp1** SAS2016	CD91	California, USA	grape	KX263137	KX263090
***Xiphinema* sp1** SAS2016	CD95	California, USA	grape	KX263133	KX263091
***Xiphinema* sp1** SAS2016	CD96	California, USA	grape	KX263123	KX263089
***Xiphinema* sp1** SAS2016	CD139	California, USA	walnut	KX263130	KX263092
***Xiphinema* sp1** SAS2016	XA13	California, USA	alfalfa	KX263126	KX263097
***Xiphinema* sp1** SAS2016	XA19	California, USA	alfalfa	KX263120	KX263099
***Xiphinema* sp1** SAS2016	XA26	California, USA	plum	KX263124	KX263101
***Xiphinema* sp1** SAS2016	XA65	California, USA	plum	KX263121	KX263085
***Xiphinema* sp1** SAS2016	XA100	California, USA	walnut	KX263122	KX263088
Xiphinema tarjanense	-	USA	Orchard fruit	-	AM086694
71. ***Xiphinema* sp2** SAS2016	CD152	California, USA	orange	KX263167	KX263105
72. ***Xiphinema* sp5** SAS2016	XA88	California, USA	peach	-	KX263045
Genus ***Longidorus*** spp.					
73. ***Longidorus aetneus***	CD1138	Varenikovskaya, Krymsk, Krasnodar Terr., Russia	Silver poplar	KF242324	KY816655
Longidorus aetneus	CD1121	Varenikovskaya, Krymsk, Krasnodar Terr., Russia	*Populus* sp.	KF242323	KY816656
Longidorus aetneus	CD1111	Varenikovskaya, Krymsk, Krasnodar Terr., Russia	*Salix fragilis*	KF242318	KY816657
Longidorus aetneus	CD1129	Varenikovskaya, Krymsk, Krasnodar Terr., Russia	*Acer tataricum*	KF242321	KY816658
Longidorus aetneus	CD1142	Varenikovskaya, Krymsk, Krasnodar Terr., Russia	*Salix alba*	KF242322	KY816659
74. ***Longidorus africanus***	P00011	Chott-mariem province, Tunisia	cultivated olive	KX062665	KY816660
75. ***Longidorus alvegus***	ALNOR	Andújar, Jaén province, Spain	black alder	*	KY816661
76. ***Longidorus andalusicus***	J0172	Sanlúcar de Barrameda, Cádiz province, Spain	pickle weed	JX445118	KY816662
77. ***Longidorus apulus***	BARLE	Barletta, Bari province, Italy	artichoke	AY601571	KY816663
78. ***Longidorus artemisiae***	CD1127	Shestikhino, Myshkin district, Yaroslavl, Russia	*Poa* sp.	KF242314	KY816664
79. ***Longidorus asiaticus***	LARGE	Bari province, Italy	crape myrtle	KR351254	KY816665
80. ***Longidorus baeticus***	M0121	Montemayor, Córdoba province, Spain	grapevine	JX445106	KY816666

Nematode species	Sample code	Locality	Host plant	GenBank accession numbers	
81. ***Longidorus closelongatus***	23CRE	Mires, Heraklion Province, Crete, Greece	grapevine	KJ802865	KY816667
82. ***Longidorus crataegi***	M0156	Montemayor , Córdoba province, Spain	grapevine	JX445114	KY816668
Longidorus crataegi	M0156	Montemayor , Córdoba province, Spain	grapevine	*	KY816669
83. ***Longidorus cretensis***	TOCRE	Pentamodi, Heraklion province, Crete, Greece	cultivated olive	KJ802868	KY816670
84. ***Longidorus distinctus***	CD1128	Pyatigorsk, Stavropol Territory, Russia	*Salix sp.*	KF242317	KY816671
85. ***Longidorus euonymus***	CD1118	Bolshoy Vyas, Lunino district	*Asparagus cicer*	KF242333	KY816672
Longidorus euonymus	CD1130	Anapa, Anapa district, Krasnodar Territory, Russia	*Juglans regia*	KF242332	KY816673
86. ***Longidorus fasciatus***	M0063	Monturque , Córdoba province, Spain	grapevine	JX445108	KY816674
87. ***Longidorus helveticus***	-	Stari Ledinci, Serbia	Unknown	EF538753	EF538747
Longidorus helveticus	-	Chodovlice, Czech republic	Sweet cherry	JN627414	JN627416
Longidorus helveticus	-	Silnicna, Czech republic	*Prunus avium* L.	JN627415	JN627417
88. ***Longidorus indalus***	ST042	Las Tres Villas, Almería province, Spain	cultivated olive	KT308854	KY816675
89. ***Longidorus intermedius***	CD1122	Kamennomostsky, Adygeya, Russia	*Fagus orientalis*	KF242312	KY816676
90. ***Longidorus iranicus***	GRECD	Harakas province, Greece	grapevine	KJ802875	KY816677
91. ***Longidorus iuglandis***	H0183	Bonares, Huelva province, Spain	grapevine	JX445104	KY816678
92. ***Longidorus jonesi***	MIY03	Miyazaki, Japan	*Prunus* sp.	KF552069	KY816679
93. ***Longidorus kuiperi***	BOLOI	Bolonia, Cádiz province, Spain	marram grass	*	KY816680
94. ***Longidorus laevicapitatus***	ACC01	La Virgen de Sarapiquí, Heredia, Costa Rica	Sugar cane	KX136865	KY816681
95. ***Longidorus leptocephalus***	CD1136	Potrosovo, Kozelsk district, Kaluga region, Russia	common nettle	KF242326	KY816682
96. ***Longidorus lignosus***	CD1124	Sukko, Anapa district, Krasnodar, Territory, Russia	*Acer campestre*	KF242345	KY816683
97. ***Longidorus lusitanicus***	J0212	Sanlúcar de Barrameda, Cádiz province, Spain	wild olive	KT308869	KY816684
98. ***Longidorus macrodorus***	JAO06	La Grajuela (Córdoba, Spain)	cultivated olive	KT308855	KY816685
Longidorus macrodorus	JAO06	La Grajuela (Córdoba, Spain)	cultivated olive	KT308856	KY816686
99. ***Longidorus macrosoma***	-	Orth, Austria	Unknown	EF538752	EF538746
100. ***Longidorus magnus***	M0130	Hinojos, Huelva province, Spain	cultivated olive	KX00000	KY816687
Longidorus magnus	M0079	Monturque, Córdoba province, Spain	grapevine	KX00000	KY816688
Longidorus magnus	M0017	Lucena, Córdoba province, Spain	grapevine	JX445113	KY816689
Longidorus magnus	J0164	Jerez de la Frontera, Cádiz, Spain	grapevine	*	KY816690
Longidorus magnus	ST077	Espiel, Córdoba province, Spain	cultivated olive	*	KY816691
Longidorus magnus	JAO01	Villaviciosa, Córdoba province, Spain	cultivated olive	*	KY816692
Longidorus magnus	JAO31	Antequera, Málaga province, Spain	cultivated olive	*	KY816693
Longidorus magnus	CASLO	Castillo de Locubin, Granada province, Spain.	cherry tree	*	KY816694
101. ***Longidorus onubensis***	ST005	Niebla (Huelva, Spain)	cultivated olive	KT308857	KY816695
102. ***Longidorus orientalis***	-	Murcia, Spain	Grapevine	KP406931	KP406950
Longidorus orientalis	CD1553	Arizona, USA	*Phoenix dactylifera*	*	KP406952
Longidorus orientalis	CD1537	California, USA	*Phoenix dactylifera*	*	KP406957
Longidorus orientalis	R0044	El Rocío, Huelva province, Spain	Gramineae	GU001823	KP406961
Longidorus orientalis	CD1562	Khuzestan Province, Ahvaz, Iran	*Phoenix dactylifera*	KJ802877	KP406962
Longidorus orientalis	CD1563	Khuzestan Province, Ahvaz, Iran	*P. dactylifera*	-	KP406964

Nematode species	Sample code	Locality	Host plant	GenBank accession numbers	
Longidorus orientalis	CD1564	Khuzestan Province, Ahvaz, Iran	*P. dactylifera*	-	KP406965
Longidorus orientalis	O44	Heraklion Province, Crete, Greece	grapevine	KJ802877	KP406963
Longidorus orientalis	P28	Alhama de Murcia, Spain	grapevine	KP406931	KP406951
Longidorus orientalis	N10	Bollullos par del Condado, Huelva, Spain	grapevine	KP406933	KP406949
103. ***Longidorus persicus***	ESMAE	Gilan-e-Gharb, Kermanshah province, Iran	rose	KT149799	KY816696
104. ***Longidorus pisi***	0IRAN	Markazi province, Iran	apple tree	JQ240274	KY816697
105. ***Longidorus poessneckensis***	-	Velke Kapusany, Slovakia	Unknown	EF538751	EF538745
Longidorus poessneckensis	-	Černé Voděrady, Czech Republic	*Quercus*	EF538750	EF538744
106. ***Longidorus pseudoelongatus***	AR034	Voutes province, Greece	cultivated olive	KJ802870	KY816698
Longidorus pseudoelongatus	AR040	Hersonisos province, Greece	cultivated olive	KJ802871	KY816699
107. ***Longidorus rubi***	H0026	Almonte, Huelva province, Spain	Pinus pinea	JX445116	KY816700
108. ***Longidorus silvestris***	AR027	Bolonia (Cádiz, Spain)	cultivated olive	KT308859	KY816701
109. ***Longidorus uroshis***	-	Velke Pole. Slovakia	Unknown	EF538754	EF614264
110. ***Longidorus vallensis***	AR055	San José del Valle, Cádiz province, Spain	wild olive	KT308861	KY816702
Longidorus vallensis	M0012	Cabra, Córdoba province, Spain	grapevine	KT308862	KY816703
111. ***Longidorus vineacola***	AR031	Tarifa, Cádiz province, Spain	wild olive	KT308873	KY816704
Longidorus vineacola	AR113	Alcolea, Córdoba province, Spain	wild olive	*	KY816705
Longidorus vineacola	TRASI	Córdoba province, Spain	cultivated olive	*	KY816706
Longidorus vineacola	M0124	Montemayor, Córdoba province, Spain	Portuguese oak	*	KY816707
Longidorus vineacola	M0124	Montemayor, Córdoba province, Spain	Portuguese oak	*	KY816708
Longidorus vineacola	0419B	Andújar, Jaen province, Spain	wild olive	*	KY816709
Longidorus vineacola	H0089	Almonte, Huelva province, Spain	Stone pine	*	KY81671
Longidorus vineacola	SR117	Setenil de las Bodegas, Cádiz province, Spain	cultivated olive	*	KY816711
Longidorus vineacola	ST016	El Saucejo, Sevilla province, Spain	cultivated olive	KT308872	KY816712
112. ***Longidorus vinearum***	AR097	Santa Mª de Trassierra (Córdoba, Spain)	wild olive	KT308876	KY816713
113. ***Longidorus wicuolea***	AR0101	Bonares (Huelva, Spain)	wild olive	KT308865	KY816714
114. ***Longidorus* sp.3**	CD1112	Natukhaevskaya, Krasnodar Territory, Russia	*Prunus divaricata*	KF242335	KY816715
115. ***Longidorus* sp.4**	CD1117	Proletarka, Krasnosulinsk, Rostov region, Russia	*Salix babylonica*	KF242334	KY816716
116. ***Longidorus* sp.6**	CD876	Point Reyes, Marin county, California, USA	unknown	KF242328	KY816717
Genus ***Paralongidorus*** spp.					
117. ***Paralongidorus bikanerensis***	BAMIR	Bam, Kerman province, Iran	Palm	JN032584	KY816718
118. ***Paralongidorus iranicus***	NOURI	Nour, Mazandaran province, Iran	Pine	JN032587	KY816719
119. ***Paralongidorus litoralis***	ZAHAR	Zahara de los Atunes, Cádiz province, Spain	mask tree	EU026155	KY816720
120. ***Paralongidorus paramaximus***	ALGUC	Alcalá de Guadaira, Sevilla province, Spain	citrus	EU026156	KY816721
Paralongidorus paramaximus	ALGUC	Alcalá de Guadaira, Sevilla province, Spain	citrus	*	KY816722
Paralongidorus paramaximus	ALGUC	Alcalá de Guadaira, Sevilla province, Spain	citrus	*	KY816723

[1]For species identification see: [9,19,20,25,27,40,41,44,45,46,47,48,63,64,65,66,67,68,69].

[2](*) Sequenced population but not deposited in GenBank database, since was identical to other sequences of the same species already deposited in GenBank.

[3](-) Sequence not obtained or not performed.

Table S2. Molecular interspecific variability for *coxI* gene within the studied genera (*Xiphinema*, *Longidorus* and *Paralongidorus*).

	Number of sequences	Similarity[1] (%)	(%) Singletones	(%) Parsimony informative sites
**Genus *Xiphinema* spp.**				
Xiphinema americanum-group				
1. X. americanum[2]	22	78.82	13.23	8.23
2. X. brevicolle 'complex'[3]	14	76.67	0.28	23.64
3. X. browni	3	99.22	0.53	0.00
4. X. californicum	18	89.83	5.08	5.08
5. X. floridae	2	98.52	-	-
6. X. incognitum	2	86.61	-	-
7. X. opisthohysterum	4	95.49	4.01	0.00
8. X. pachtaicum	21	94.85	1.63	1.90
9. X. peruvianum[5]	2	79.71	-	-
10. X. rivesi	7	78.09	6.74	15.17
11. X. simile	6	94.31	4.95	0.74
12. X. vallense	2	100.00	-	-
13. Xiphinema sp.1	18	82.26	8.39	9.35
14. Xiphinema sp. JPN-2014	2	100.00	-	-
Xiphinema non-*americanum group*				
15. X. adenohystherum	5	88.40	10.90	3.94
16. X. andalusiense	3	97.20	2.81	0.00
17. X. baetica	2	96.90	-	-
18. X. bakeri	2	97.49	-	-
19. X. celtiense	2	93.97	-	-
20. X. chambersi	8	98.62	0.92	0.46
21. X. costaricense	2	99.11	-	-
22. X. coxi europaeum	2	93.10	-	-
23. X. diversicaudatum	29	91.80	1.64	5.74
24. X. herakliense	3	96.90	2.62	0.00
25. X. hispidum	2	95.95	-	-
26. X. index	9	98.34	0.95	-
27. X. italiae	6	69.73	9.93	20.34
28. X. krugi	2	96.76	-	-
29. X. lupini	3	98.50	1.50	0.00

30. X. mengibarense	2	99.46	-	-
31. X. nuragicum	5	95.79	1.32	2.89
32. X. setariae	2	100.00	-	-
**Genus *Longidorus* spp.**				
33. L. aetnaeus	5	99.37	0.63	0.00
34. L. crataegi	2	98.41	-	-
35. L. euonymus	2	100.00	-	-
36. L. helveticus	3	92.39	7.61	0.00
37. L. macrodorus	2	99.68	-	-
38. L. magnus	8	78.70	3.97	17.33
39. L. orientalis	17	78.78	0.72	20.50
40. L. pseudoelongatus	2	100.00	-	-
41. L. poessneckensis	2	84.62	-	-
42. L. vallensis	2	100.00	-	-
43. L. vineacola	9	68.91	7.05	24.04
Paralongidorus sp.				
44. P. paramaximus	2	99.31	-	-

[1]Minimum similarity percentage
(-) Not determined
[2] *X. americanum cf.* not included
[3]*X. brevicolle* 'complex'*: X. diffusum, X. taylori, X. brevicolle,* (not include accession AM086707 probably wrong identity) and not included in further analysis

Table S3. Molecular interspecific variability for D2-D3 region within the studied genera (*Xiphinema, Longidorus* and *Paralongidorus*).

	Number of sequences	Similarity (%)	(%) Singleton bases	(%) Parsimony informative sites
**Genus *Xiphinema* spp.**				
Xiphinema americanum-group				
1. X. americanum	20	94.65	3.67	1.53
2. X. brevisicum	2	99.83	-	-
*3. X. brevicolle**	21	97.41	1.59	1.01
4. X. bricolensis	2	99.60	-	-
5. X. browni	4	100.00	0.00	0.00
6. X. californicum	17	95.69	2.72	1.59
7. X. citricolum	6	100.00	0.00	0.00
8. X. duriense	2	99.58	-	-
9. X. floridae	4	100.00	0.00	0.00
10. X. georgianum	9	99.73	0.27	0.00
11. X. incertum	5	99.47	0.27	0.27
12. X. laevistriatum	4	99.76	0.24	0.00
13. X. opisthohysterum	2	100.00	-	-
14. X. pachtaicum	25	96.51	1.90	1.27
15. X. parapachydermum	6	98.73	0.79	0.47
16. X. plesiopachtaicum	3	100.00	0.00	0.00
17. X. rivesi	23	95.72	0.14	2.48
18. X. santos	3	100.00	0.00	0.00
19. X. simile	7	99.18	0.41	0.27
20. X. thornei	2	99.73	-	-
21. X. vallense	3	100.00	0.00	0.00
22. Xiphinema sp. 1 SAS-2016	23	98.40	0.89	0.89
23. Xiphinema sp. 3 SAS-2016	2	100.00	-	-
24. Xiphinema sp. 4 SAS-2016	2	99.71	-	-
Xiphinema non-*americanum group*				
25. X. adenohystherum	5	99.02	0.84	0.14
26. X. andalusiense	5	99.49	0.51	0.00
27. X. baetica	6	98.71	0.71	0.57
28. X. bakeri	5	98.69	1.05	0.26
29. X. barense	3	99.72	0.28	0.00

30.	*X. basiri*	2	100.00	-	-
31.	*X. belmontense*	3	100.00	-	-
32.	*X. brasiliense*	2	100.00	-	-
33.	*X. celtiense*	2	99.31	-	-
34.	*X. chambersi*	9	97.44	0.71	0.71
35.	*X. cohni*	2	97.00	-	-
36.	*X. conurum*	4	99.39	0.60	0.00
37.	*X. costaricense*	4	99.68	0.16	0.16
38.	*X. coxi europaeum*	7	99.73	0.28	0.00
39.	*X. cretense*	4	99.73	0.27	0.00
40.	*X. dentatum*	2	99.87	-	-
41.	*X. diversicaudatum*	13	98.90	0.54	0.54
42.	*X. elongatum*	4	99.61	0.00	0.39
43.	*X. herakliense*	8	99.34	0.47	0.16
44.	*X. hispidum*	4	99.15	0.85	0.00
45.	*X. hunaniense*	9	97.93	0.39	1.68
46.	*X. index*	19	99.42	0.29	0.29
47.	*X. ingens*	2	99.59	-	-
48.	*X. israeliae*	4	99.43	0.43	0.14
49.	*X. italiae*	10	97.05	1.12	1.83
50.	*X. krugi*	4	100.00	0.00	0.00
51.	*X. lupini*	2	99.85	-	-
52.	*X. macroacanthum*	2	96.56	-	-
53.	*X. macrodora*	6	99.86	-	-
54.	*X. mengibarense*	3	99.86	0.14	0.00
55.	*X. meridianum*	3	99.73	0.41	0.00
56.	*X. nuragicum*	10	100.00	0.00	0.00
57.	*X. oleae*	3	99.32	0.68	0.00
58.	*X. pseudocoxi*	2	99.67	-	-
59.	*X. robbinsi*	3	99.38	0.41	0.00
60.	*X. setariae*	6	98.86	0.85	0.28
61.	*X. sphaerocephalum*	2	99.47	-	-
62.	*X. turcicum*	4	99.47	0.40	0.13
63.	*X. turdetanense*	2	99.46	-	-
64.	*X. vuittenezi*	5	99.47	0.26	0.26
65.	*Xiphinema* sp. P11	3	100.00	0.00	0.00

**Genus *Longidorus* spp.**				
66. L. aetnaeus	10	99.31	0.55	0.14
67. L. africanus	10	98.42	1.29	0.28
68. L. andalusicus	3	99.71	0.29	0.00
69. L. artemisiae	4	99.85	0.14	0.00
70. L. alvegus	4	99.02	0.84	0.14
71. L. asiaticus	3	99.06	0.80	0.00
72. L. attenuatus	3	100.00	0.00	0.00
73. L. baeticus	2	98.45	-	-
74. L. caespiticola	5	95.97	2.92	0.42
75. L. carniolensis	2	100.00	-	-
76. L. cholevae	2	100.00	-	-
77. L. closelongatus	5	100.00	0.00	0.00
78. L. cretensis	3	99.58	0.42	0.00
79. L. danuvii	2	100.00	0.00	0.00
80. L. distinctus	2	100.00	0.00	0.00
81. L. elongatus	19	98.70	1.00	0.29
82. L. euonymus	5	99.59	0.41	0.00
83. L. helveticus	5	99.46	0.54	0.00
84. L. henanus	2	99.73	-	-
85. L. glycines	3	98.97	1.03	0.00
86. L. indalus	3	99.70	0.30	0.00
87. L. intermedius	4	98.56	1.01	0.43
88. L. iranicus	5	99.86	0.13	0.00
89. L. iuglandis	2	100.00	-	-
90. L. juvenilis	2	99.03	-	-
91. L. kuiperi	2	100.00	-	-
92. L. laevicapitatus	4	100.00	0.00	0.00
93. L. leptocephalus	5	99.57	0.29	0.00
94. L. lignosus	3	100.00	-	-
95. L. magnus	5	99.58	0.42	0.00
96. L. macrodorus	2	100.00	-	-
97. L. macrosoma	2	99.72	-	-
98. L. oleae	2	99.71	0.29	0.00
99. L. onubensis	2	100.00	-	-
100. L. orientalis	7	99.57	0.28	-

101.L. persicus	2	100.00	-	-
102.L. pisi	3	99.58	0.42	0.00
103.L. pseudoelongatus	4	99.86	0.14	0.00
104.L. poessneckensis	2	100.00	-	-
105.L. silvestris	2	100.00	-	-
106.L. vallensis	2	99.71	-	-
107.L. vineacola	5	98.30	1.55	0.14
108.L. vinearum	4	99.45	0.55	0.00
109.L. wicuolea	4	99.15	0.85	0.00
110.Longidorus sp. 5 2014	2	99.73	-	-
111.*Longidorus* sp. JH2014	3	100.00	0.00	0.00
**Genus *Paralongidorus* spp.**				
112.P. rex	5	99.85	0.15	0.00

(-) Not determined

X. brevicolle ‘complex’*: X. diffusum, X. taylori*, *X. brevicolle* and *X. inaequale*, not included in further analysis

Table S4. Molecular interspecific variability for *ITS* region within the studied genera *(Xiphinema, Longidorus* and *Paralongidorus*)*.

	Nº seq.	Similarity %	Singleton sites %	Parsimony informative sites %		Nº seq.	Similarity %	Singleton sites %	Parsimony informative sites %
**Genus *Xiphinema* spp.**									
Xiphinema americanum group					*Xiphinema* non-*americanum* group				
1. X. americanum	37	78.61	13.70	5.68	*14. X. andalusiense*	6	97.66	0.49	1.65
2. X. brevicolle	7	92.10	2.46	4.02	*15. X. baetica*	2	98.92	0.00	0.00
3. X. browni	3	100.00	0.00	0.00	*16. X. bakeri*	3	97.49	1.99	0.00
4. X. citricolum	4	97.87	1.35	0.14	*17. X. bernardi*	3	99.49	0.51	0.00
5. X. floridae	4	97.38	1.79	0.07	*18. X. brasiliense*	2	89.21	-	-
6. X. georgianum	8	96.78	1.75	1.33	*19. X. celtiense*	2	96.73	-	-
7. X. laevistriatum	4	98.67	0.62	0.00	*20. X. chambersi*	11	87.22	6.47	3.99
8. X. inaequale	2	79.15	-	-	*21. X. conurum*	2	98.56	-	-
9. X. oxycaudatum	2	100.00	0.00	0.00	*22. X. costaricense*	4	93.49	4.43	0.55
10. X. rivesi	9	93.02	1.98	4.10	*23. X. coxi europaeum*	3	100.00	0.00	0.00
11. X. pachtaicum	3	91.32	1.30	0.00	*24. X. diversicaudatum*	4	96.57	1.55	1.33
12. X. santos	2	100.00	-	-	*25. X. herakliense*	3	99.22	0.67	0.00
13. X. simile	2	100.00	-	-	*26. X. hunaniense*	2	97.73	-	-
					27. X. index	5	99.03	0.68	0.19
					28. X. ingens	2	98.85	-	-
					29. X. insigne	2	94.86	-	-
					30. X. italiae	8	89.91	5.23	2.88
					31. X. iznajarense	2	99.09	-	-
					32. X. krugi type A	2	94.12	-	-
					33. X. krugi type B	3	90.26	6.46	0.00
					34. X. krugi type C	5	93.03	4.44	0.89
					35. X. macrodora	3	99.42	0.58	0.00
					36. X. mengibarense	2	97.72	-	-
					37. X. nuragicum	3	99.70	0.19	0.00
					38. X. oleae	2	99.55	-	-
					39. X. pseudocoxi	2	97.15	-	-
					40. X. setariae	3	99.16	0.82	0.00

	Nº	Conserved	Singleton	Parsimony

	seq.	sites %	sites %	informative sites %
**Genus *Longidorus***				
41. L. africanus	2	98.06	-	-
42. L. arthensis	2	99.56	-	-
43. L. asiaticus	2	99.12	-	-
44. L. biformis	3	84.59	4.20	9.11
45. L. caespiticola	2	99.75	-	-
46. L. diadecturus	2	98.86	-	-
47. L. elongatus	4	99.31	0.49	0.00
48. L. euonymus	2	99.99	-	-
49. L. glycines	3	92.73	4.69	0.00
50. L. indalus	2	97.71	-	-
51. L. iranicus	2	99.80	-	-
52. L. kuiperi	13	93.13	1.74	4.97
53. L. laevicapitatus	4	99.34	0.33	0.11
54. L. macrodorus	2	99.49	-	-
55. L. orientalis	2	96.92	0.40	1.60
56. L. onubensis	2	100.00	-	-
57. L. pius	8	99.86	0.00	0.13
58. L. raskii	2	99.24	-	-
59. L. vallensis	2	97.08	-	-
60. L. vinearum	2	100.00	-	-
61. L. wicuolea	3	99.46	0.40	0.00
62. Longidorus sp. JH2014	2	100.00	-	-
63. Longidorus sp. M30	2	99.01	-	-
Genus Paralongidorus				
64. P. rex	4	99.43	0.85	0.00

*Some sequences of *X. diversicaudatum* have been corrected following Chizhov *et al.*[18]

***coxI* phylonetic tree Newick format**. Phylogenetic relationships within Longidoridae. Bayesian 50% majority rule consensus tree as inferred from the analysis of the partial coxI sequence alignment under a TrN + I+ G model. Posterior probabilities more than 0.70 are given for appropriate clades.

(KC618473Helachista:0.9965429,KP718973_Rstriaticeps:0.6348314,((((((((((nemv53Xadenohysterum_coi:0.02323174,nemn42Xadenohysterum_coi:0.04442051):0.03462923,((AR86adenohystherum_t9:0.02441121,NBTadenohystherum_AA74:0.02143105):0.01531369,ar78adenohystherum_P6:0.04497179):0.04875309):0.2324454,((nemM51XitaliaeAR41:0.1722289,(APULIA_ITALIAE:0.08117989,FJ713151XiphinemaitaliaeITA:0.08723101):0.1084252):0.1594631,(nemT45XitaliaeAR91:0.1109185,(Q44italiae_tunez:0.02038836,R10italiae_tunez:0.04536965):0.08883677):0.1655259):0.1725677,J126Xcohni:0.2808372,708Xhispanum:0.2669905,(nem875Xhispidum:0.0346082,nemU75Xhispidum:0.02400078):0.3062302,((Xceltiense_ar82:0.07003489,ar83Xceltiense:0.04415578):0.1903435,(K32Xmengibarense:0.002395316,Xmengibarense_K42:0.008163319):0.5238048):0.103768,Xiznajarense_JAO25_U50:0.3134576):0.1290071,N41Xpyrenaicum:0.316702,N40Xsphaerocephalum:0.2617255,(((nemL68_Xnuragicum_ST12C:0.002950092,JAO28Xnuragicum:0.002681987):0.01108944,(XnuragicumAR113:0.03190949,(AR54Xnuragicum:0.002460877,ST106Xnuragicum:0.002595045):0.0325636):0.02782069):0.2828854,((NEMn30_Xherakliense_oleaster18:0.004528226,nemN29X_herakliense_oleaster17:0.0278936,NEMM64X_herakliense_oleaster8:0.002248172):0.2218904,nemN28X_israeliae_AR16delgadoG:0.5221968):0.1861949):0.1843163):0.1438582,(XconurumST45V_NEMN4_nuevo:0.4807968,Xmeridianum_q31:0.30496):0.09090819,XrobbinsinemZ21:0.5752856):0.1806537,((((X_belmontense_mouchoDario:0.3492614,(AR20nemL26Xcoxieuropeaum:0.03020569,H27Xcoxieuropaeum:0.03202631):0.2048132):0.1273966,EU781537Xiphinemadentatum:0.5031269):0.1167588,(((ar93andalusiense_t60:0.01180015,(419andalusiense_o57:0.005733587,ar108andalusiense_v14:0.01005144):0.02095353):0.2935354,((nem770Xbaetica_coi:0.02442197,nem791Xbaetica_coi:0.02705422):0.07283846,H59Xgersoni:0.08074309):0.1785847):0.05074762,JAO47_MACRODORA_NEMZ11:0.2309812):0.1256444,Xcadavalense_st77_p49:0.2135329,Italy_Xmacroancthum_aa54:0.3368099,((((((KF292284Xiphinemadiversicaudat:0.002351812,KF292285Xiphinemadiversicaudat:0.002484021,KF292286Xiphinemadiversicaudat:0.002343647,KF292287Xiphinemadiversicaudat:0.002352577,KF292288Xiphinemadiversicaudat:0.00264783,KF292289Xiphinemadiversicaudat:0.002320051,KF292290Xiphinemadiversicaudat:0.002387069,KF292291Xiphinemadiversicaudat:0.002435328,KF292292Xiphinemadiversicaudat:0.005807669,(KF292293Xiphinemadiversicaudat:0.002399442,KF292294Xiphinemadiversicaudat:0.005859068):0.00601303):0.006742832,(JQ780331Xiphinemadiversicaudat:0.002246298,EF538749Xiphinemadiversicaudat:0.005376788):0.00535917):0.01297236,(KF292295Xiphinemadiversicaudat:0.002451813,KF292296Xiphinemadiversicaudat:0.002426701,KF292297Xiphinemadiversicaudat:0.002387624):0.008733526,KF292298Xiphinemadiversicaudat:0.002406784,KF292299Xiphinemadiversicaudat:0.002443191,KF292302Xiphinemadiversicaudat:0.00580435,KF292300Xiphinemadiversicaudat:0.005855249,KF292301Xiphinemadiversicaudat:0.00950027,((KF292304Xiphinemadiversicaudat:0.002373756,KF292303Xiphinemadiversicaudat:0.002467526):0.01439617,GU222423Xiphinemadiversicaudat:0.007884835):0.008663025):0.008865756,(JQ780332Xiphinemadiversicaudat:0.002286925,JQ780333Xiphinemadiversicaudat:0.002244985,GU222422Xiphinemadiversicaudat:0.0021879):0.01561524):0.01194666,(JQ780330Xiphinemadiversicaudat:0.002357271,GU222421Xiphinemadiversicaudat:0.002434923):0.009106993):0.1808502,(KF292305XiphinemabakeriCD852:0.004714711,KF292306XiphinemabakeriCD840:0.01486429):0.07421353):0.1613755,(nemP58Xcretense_COI:0.3895626,(HM921380Xiphinemaindexnem75:0.002213138,HM921381Xiphinemaindexnem89:0.002443212,HM921383Xiphinemaindexnem94:0.002436054,HM921384Xiphinemaindexnem95:0.002334589,HM921385Xiphinemaindexnem96:0.002315738,HM921386Xiphinemaindexnem97:0.002337561,HM921387Xiphinemaindexnem98:0.005401451,HM921388Xiphinemaindexnem99:0.002260069,HM921382Xiphinemaindexnem90:0.005539164):0.4004788):0.1234915,Xpseudocoxi_ar95:0.288131,AR15Xturdetanense:0.3270409):0.1617674,(((KU764412Xiphinemachambersiisol:0.002352217,KU764413Xiphinemachambersiisol:0.002439921,KU764414Xiphinemachambersiisol:0.002432345,KU764415Xiphinemachambersiisol:0.002488717,KU764416Xiphinemachambersiisol:0.005675138,KU764417Xiphinemachambersiisol:0.006055713,KU764418Xiphinemachambersiisol:0.005786994,KU764419Xiphinemachambersiisol:0.005585411):0.2982692,(KX263044Xiphinemasetariaeisola:0.002800263,setariae_ac56:0.002734007):0.7724071):0.1126419,(((Xinsigne_cementerioJapom:0.3274354,((lupini_nem722:0.00206226,nem723lupini:0.002024357):0.006555245,nem865_LUPINI:0.0187705):0.337864):0.1230506,XSPTUNEZ:0.2969175):0.178997,Xturcicum_ST149:0.4108596):0.14295):0.04389835):0.0837731):0.0885848,((XKRUGI_AB19:0.03255635,XKRUGIR80:0.02341312):0.4912763,(ACC61xCOSTARICENSE:0.01298203,ACC46xCOSTARICENSE:0.00408777):0.5014219):0.174966):0.06277527,((((v89Pparamaximus:0.002175349,v91Pparamaximus:0.002193049,E89Pparamaximus:0.002232569):0.4029746,COX1_PLITO:0.4573376):0.149335,PiranicusnemE80:0.2897602):0.4495109,(KX263045Xiphinemasp5SAS2016iso:0.426085,LjonesiL5:0.80298):0.1603869,(((nemo61LalvegusDIFERENTEanterio:0.3848032,((Lcloselongatus:0.2106982,(LkuiperiZ:0.

5633112,(Lmoesicus100:0.181209,(NEMN23Lpseudoelongatus:0.002169106,NEMN24Lpseudoelongatu
s:0.002073607):0.2336349):0.1726696):0.1883645):0.146142,(LcretensisnemO45:0.2104319,CD1117Lo
ngidorussp4HR1SB439:0.1988107):0.2156916):0.2370033):0.09664141,((CD1111LaetnaeusHR1SB442:
0.002548526,CD1143LaetnaeusHR1SB444:0.002569358,CD1129LaetnaeusHR1SB445:0.002490262,C
D1138LaetnaeusHR1SB446:0.006160113,CD1108LaetnaeusHR1SB441:0.005560582):0.1858626,CD11
19LleptocephalusHR1SB4:0.4101035):0.2317798,((CD1118LeuonymusHR1SB410:0.002461023,CD113
0LeuonymusHR1SB443:0.002589172):0.3181338,nemN64LapulusBurletta:0.2977682):0.1365054,Linda
lusnemM47:0.5004061,CD1112Longidorussp3HR1SB409:0.5132525,Lpersicus:0.6479486):0.1340517,(
((NEMC81Lbaeticus:0.2393001,((LmacrodorusnemV33:0.003646684,LmacrodorusnemV4:0.008829639
):0.3384303,LfasciatusNEMF25:0.329179):0.09411689):0.1170235,(nemN62Landalusicus:0.2620771,((
KP406949LongidorusorientalisN1:0.002785276,KP406950LongidorusorientalisN1:0.002865979,KP4069
51LongidorusorientalisP2:0.002746057):0.1110561,((KP406952LongidorusorientalisCD:0.002776491,K
P406953LongidorusorientalisCD:0.002740524,KP406954LongidorusorientalisCD:0.002880949,KP4069
55LongidorusorientalisCD:0.002840653):0.02953317,KP406956LongidorusorientalisCD:0.03507444,(((
KP406957LongidorusorientalisCD:0.002897674,KP406958LongidorusorientalisCD:0.002793328,KP406
959LongidorusorientalisCD:0.002853263,KP406960LongidorusorientalisCD:0.002805676):0.006525554
,KP406961LongidorusorientalisR4:0.003872311):0.01100494,KP406962LongidorusorientalisCD:0.0028
44225,KP406963LongidorusorientalisO4:0.002910205,KP406964LongidorusorientalisCD:0.002957754,
KP406965LongidorusorientalisCD:0.00688595):0.2100842):0.05956746):0.1602006):0.2222198):0.1170
247,(((M156v23Lcrataegi:0.008554521,Lcrataegim156:0.02077787):0.3434466,LlusitanicusnemK2:0.29
72574):0.1084609,(((((jao31magnus:0.04094327,m130Lmagnus:0.07146728):0.09939742,(castilloMagn
us:0.004493309,M79MAGNUS:0.0023477,M17MAGNUS:0.002845582,J164MAGNUS:0.007867522):
0.1240988,(ST77MAGNUS:0.002379036,JAO1MAGNUS:0.002354669):0.1545686):0.1736596,Lvinear
umnemU69:0.2329601):0.08778895,Lonubensisnemw31:0.3565027):0.1246609,(((st16vineacolaJB3:0.0
8660072,AR31vineacolaJB3:0.1684391,ST117vineacolaCOIF:0.1604709,(TRASIERRAvineacolaCOIF:
0.1082963,(419bvineacola:0.13077,(M124JB3vineacola:0.002263904,M124Lvineacola:0.002262322):0.
09155735):0.1048071):0.05216688):0.1063863,(F23vineacolaCOIF:0.002144992,H89Lvineacola:0.0021
43855):0.1754024):0.1406868,(LsilvestrisnemX11:0.2261833,LwicuoleanemW16:0.2960614):0.3119236
):0.09852643):0.0784343):0.05182939,nemE61Liuglandis:0.4459177):0.1458026,((LvallensisN35:0.002
302574,LSPXm12_VALLENSIS:0.002341827):0.3399111,LrubiH26:0.4488914):0.2001546,((CD1127L
artemisiaeHr1SB414:0.2645588,(CD1122LintermediusHR1SB447:0.404661,EF614264Longidorusuroshi
sfromS:0.3358539):0.1082117):0.1665634,(CD1128LdistinctusHr1SB413:0.3370891,CD876Longidoruss
p6HR1SB438:0.481975):0.1621367):0.08724491):0.07645721,((EF538744Longidoruspoessneckens:0.24
20956,EF538745Longidoruspoessneckens:0.1370286):0.2261627,((((EF538747Longidorushelveticusfr:0.
005710275,JN627417_Lhelveticus:0.007209524):0.04673959,JN627416_Lhelveticus:0.07102129):0.430
7002,EF538746Longidorusmacrosomafro:0.5231104):0.1458879,CD1120LlignosusHR1SB411:0.403607
7):0.1153676):0.1845633,(LpisiH57:0.5181071,LasiaticusnemJ85:0.672405):0.2135252,africanus:0.5683
882,PbikanerensisE79:0.5677614,Llaevicapitatus:0.6068479):0.0995916,((((KU250158Xiphinemabrowni
isolate:0.002695704,GU222424XiphinemabrowniPK_NSB1:0.004856798):0.004855232,AM086708Xip
hinemasimileslovaki:0.008484221):0.335738,KU250159Xiphinemaparasimileiso:0.5010631):0.3828198,
(KP268979Xiphinemabrevisicumclo:0.4273971,(KX263117Xiphinemasimileisolate:0.01123059,AM086
711Xiphinema:0.02535791,((AM086710Xiphinema:0.002508063,AM086709XiphinemacfamericanumR:
0.002272574,GU222425XiphinemasimileSC:0.002475282):0.00535519,GU222426XiphinemasimileSD:
0.002529635,GU222427XiphinemasimileSVP:0.01226633):0.05707586):0.9366808):0.1931083):0.2496
275):0.1690903,(Xduriense_ST2C:0.6025919,(XopisthoysterumAR31:0.02253686,(Xopisthoysterum418
_I94:0.005031467,JQ990054Xiphinemaopisthohyster:0.002208151,JQ990053Xiphinemaduriensevouch:0
.002066856):0.03439347):0.4811346):0.2050422,((((((((nemk43Xluci_coi:0.1231239,(KX263102Xiphin
emarivesiisolate:0.002581664,KX263103Xiphinemarivesiisolate:0.002437781):0.1650824,((AM086696
Xiphinemafloridaeflori:0.01959354,AM0866972Xiphinemarivesiarkans:0.006107392):0.285997,AM086
712Xiphinemaperuvianumbra:0.424887):0.09523526):0.09872031,KX263105Xiphinemasp2SAS2016iso
:0.1587265):0.06018926,(((((((KX263065Xiphinemaamericanumiso:0.002683438,KX263066Xiphinema
californicumi:0.005903488):0.01381376,KX263067Xiphinemacalifornicumi:0.03161097):0.00993245,(K
X263068Xiphinemacalifornicumi:0.00250344,KX263069Xiphinemacalifornicumi:0.004972248,KX2630
70Xiphinemacalifornicumi:0.002374918,KX263071Xiphinemacalifornicumi:0.005644185,KX263072Xi
phinemacalifornicumi:0.002434865,KX263074Xiphinemacalifornicumi:0.01306358,KX263073Xiphine
macalifornicumi:0.0205961):0.013432):0.0295258,KX263082Xiphinemacalifornicumi:0.00824278,KX2
63083Xiphinemacalifornicumi:0.009073921):0.008592988,KX263081Xiphinemacalifornicumi:0.009284
544):0.009496891,KX263080Xiphinemacalifornicumi:0.006266006):0.008806513,KX263075Xiphinema
californicumi:0.002279975,KX263076Xiphinemacalifornicumi:0.002380151,KX263077Xiphinemacalifo
rnicumi:0.002341786,KX263078Xiphinemacalifornicumi:0.002182207,KX263079Xiphinemacalifornicu

mi:0.00241001):0.1262001,((((((KX263084Xiphinemasp1SAS2016iso:0.007920144,KX263085Xiphine
masp1SAS2016iso:0.003862186):0.02287405,KX263086Xiphinemasp1SAS2016iso:0.03657544):0.0624
14,(((KX263087Xiphinemasp1SAS2016iso:0.005538462,KX263088Xiphinemasp1SAS2016iso:0.00273
0288,KX263093Xiphinemasp1SAS2016iso:0.01399163,KX263091Xiphinemasp1SAS2016iso:0.005781
035,(KX263092Xiphinemasp1SAS2016iso:0.007768696,(KX263094Xiphinemasp1SAS2016iso:0.00249
9211,1068088114KX263096Xiphinemasp1:0.002388471,KX263097Xiphinemasp1SAS2016iso:0.00247
0585,KX263098Xiphinemasp1SAS2016iso:0.002350728,KX263099Xiphinemasp1SAS2016iso:0.00244
2423,KX263095Xiphinemasp1SAS2016iso:0.002566347):0.006783657):0.00684099):0.005867298,KX2
63089Xiphinemasp1SAS2016iso:0.002937762):0.006224405,KX263090Xiphinemasp1SAS2016iso:0.00
2907766):0.01892058):0.02544604,AM086694Xiphinematarjanenseflo:0.02302318):0.09640226,106808
8124KX263101Xiphinemasp1:0.1232628):0.07751839,KX263104Xiphinemarivesiisolate:0.1751415,((C
astillodelocubinxrivesi:0.002549251,518Xrivesi:0.002368117):0.004670033,JQ990060Xiphinemarivesiv
oucher:0.006043402,KX263100Xiphinemarivesiisolate:0.06028306):0.1559968):0.07858703):0.0641371
8,((KX263046Xiphinemaamericanumiso:0.002471488,KX263059Xiphinemaamericanumiso:0.00255335
7,KX263048Xiphinemaamericanumiso:0.002454481,KX263060Xiphinemaamericanumiso:0.002556204,
KX263061Xiphinemaamericanumiso:0.002499852,KX263062Xiphinemaamericanumiso:0.002535819,K
X263063Xiphinemaamericanumiso:0.002507163,KX263051Xiphinemaamericanumiso:0.002644576,KX
263052Xiphinemaamericanumiso:0.002545722,KX263055Xiphinemaamericanumiso:0.002573756,KX2
63056Xiphinemaamericanumiso:0.002529109,KX263057Xiphinemaamericanumiso:0.002499919,KX26
3047Xiphinemaamericanumiso:0.01342673,KX263058Xiphinemaamericanumiso:0.002500699,(KX2630
49Xiphinemaamericanumiso:0.003399435,KX263050Xiphinemaamericanumiso:0.002528067):0.007339
121,KX263053Xiphinemaamericanumiso:0.006426677,KX263054Xiphinemaamericanumiso:0.0028186
94,AM086690Xiphinemaamericanumsou:0.005729309,AM086689XiphinemacfamericanumR:0.0023488
56,AM086691XiphinemacfamericanumR:0.01797828):0.09401317,(((KX263064Xiphinemaamericanumi
so:0.06605315,AM086692Xiphinemaperuvianumgeo:0.06972404):0.02926183,AY382608_americanum_
complete:0.0300184):0.02212563,AM086693Xiphinemacitricolumflo:0.04433387):0.05244547):0.12253
82,JQ990055Xiphinemasantosvoucher:0.1566116,AM086695Xiphinemageorgianumflo:0.2897389):0.11
82643,JQ990059Xiphinemaparabrevicoll:0.3423742):0.05580948,(((3HM163206Xiphinemabrevicollum
B:0.002353201,(AM086702Xiphinemataylori:0.002530518,117306895embAM086703Xiphinemat:0.002
314691):0.005683575):0.1212151,(KX263106Xiphinemabrevicolleiso:0.002355752,KX263107Xiphine
mabrevicolleiso:0.002442416):0.146063):0.1396046,((AB675669Xiphinemabrevicolle:0.002381644,AB
675671Xiphinemabrevicolle:0.005608013,AB604337Xiphinemabrevicolle:0.002346109,AB675670Xiphi
nemabrevicolle:0.005628651,((AM086698Xiphinemadiffusumbrazi:0.00229889,AM086699Xiphinemadi
ffusumbrazi:0.002329927,117306889embAM086700Xiphinemad:0.005614351):0.02529528,AM086701
Xiphinemadiffusumchina:0.01583555):0.008220791):0.04650781,(AB675672Xiphinemabrevicolle:0.002
321358,AB675673Xiphinemabrevicolle:0.002289413):0.07282584):0.08236114):0.09097742,(AM08670
7Xiphinemabrevicollebra:0.2873018,(AM086705Xiphinemaincognitumchi:0.1211606,AM086706Xiphin
emaincognitumchi:0.223014):0.1541364):0.1093455,((AB604338XiphinemaspJPNHS09COI:0.0027134
44,AB604339XiphinemaspJPNHS09:0.0026529):0.2743469,HM163207XiphinemainaequaleHP:0.28950
01):0.0781449):0.1670318,AM086704XiphinemacfamericanumR:0.2204155):0.1078772,HM163208Xip
hinemalambertiiXL:0.09960952):0.2513957,KP268978Xiphinemamadeirenseclo:0.7123316,(nemN53_X
vallense:0.002343751,nemL59_Xvallense:0.002275381):0.9104469):0.5276566,(((KX263108Xiphinema
pachtaicumiso:0.00240212,KX263109Xiphinemapachtaicumiso:0.002557566,KX263115Xiphinemapach
taicumiso:0.002458789,KX263110Xiphinemapachtaicumiso:0.002459326,KX263111Xiphinemapachtaic
umiso:0.002431386,KX263112Xiphinemapachtaicumiso:0.002369058,KX263113Xiphinemapachtaicumi
so:0.002397248,KX263116Xiphinemapachtaicumiso:0.01249574,KX263114Xiphinemapachtaicumiso:0.
002306202,HM921369Xiphinemapachtaicumnem:0.002287535,HM921372Xiphinemapachtaicumnem:0.
002335796,HM921373Xiphinemapachtaicumnem:0.002347973,HM921376Xiphinemapachtaicumnem:0.
002343849,HM921378Xiphinemapachtaicumnem:0.00218132,HM921375Xiphinemapachtaicumnem:0.0
03926628,HM921374Xiphinemapachtaicumnem:0.008949313,JQ990057Xiphinemapachtaicumvou:0.00
2451654):0.01914466,HM921370Xiphinemapachtaicumnem:0.008474806,(HM921371Xiphinemapachta
icumnem:0.002310352,HM921377Xiphinemapachtaicumnem:0.002343359,HM921379Xiphinemapachta
icumnem:0.002240575):0.007961255):0.09192357,JQ990058Xiphinemaincertumvouch:0.2789219):0.20
28652,JQ9900562Xiphinemaparapachyder:0.3204827,KP268977Xiphinemaastaregiensec:0.5985193):2.3
88636);

D2-D3 region phylonetic tree Newick format. Phylogenetic relationships within Longidoridae. Bayesian 50% majority rule consensus tree as inferred from the analysis of the D2-D3 region alignment under a GTR + I + G model. Posterior probabilities more than 0.70 are given for appropriate clades.

(KC618462elachista:0.3304446,KJ765343striaticeps:0.3875549,(((((((((((((((T60:0.007320508,(X_NEMO56419B:0.002974046,Xpseudocoxi419:0.002943082,X_NEMO57419B:0.002647499,AR108_28S_V14_28s:0.03053452):0.01137349):0.0354402,((AR88_Xbaetica:0.003032494,(KC567165Xbaetica:0.003003316,KC567166Xbaetica:0.007049422):0.01094237,KC567169Xbaetica:0.01062271):0.01031717,KC567167Xbaetica:0.006615035,KC567168Xbaetica:0.007076104):0.02496181):0.0104233,ST77_Xcadavalense:0.03268636):0.00988826,(5Xmacrodora:0.00286785,6Xmacrodora:0.002780554,2Xmacrodora:0.002960924,3Xmacrodora:0.002646251,4Xmacrodora:0.002787076,1Xmacrodora:0.006726694):0.05333541):0.04188505,((KU052864Xbakeri:0.006622855,DQ364685XspJZ2006:0.006756387,((KF292276Xbakeri:0.004043213,AY601623Xbakeri:0.02737743):0.006633957,KF292277Xbakeri:0.002786548,KF292278Xbakeri:0.002674916):0.01287126):0.01463208,(((HG969302Xdiversicaudatum:0.002639253,JQ780367Xdiversicaudatum:0.002820446,JQ780366Xdiversicaudatum:0.002834321,KF292279Xdiversicaudatum:0.006580599,KF292280XdiversicaudatumC:0.002759464,EF538755Xdiversicaudatum:0.02329321):0.008616778,JQ780359Xdiversicaudatum:0.003053027):0.01141815,JQ780364Xdiversicaudatum:0.002828352,JQ780363Xdiversicaudatum:0.002592501,JQ780360Xdiversicaudatum:0.00271713,JQ780365Xdiversicaudatum:0.006046967,JQ780361Xdiversicaudatum:0.002660284,JQ780362Xdiversicaudatum:0.002702052):0.01216233):0.02959312):0.01731371,(KJ634067XspSAS2014:0.01774006,AY601625Xabrantinum:0.02089432):0.03246368,((((Xcoxieuropaeum_AR92_28S:0.002894908,KC567174Xcoxieuropaeum:0.002879188,KC567175Xcoxieuropaeum:0.002905172,KC567178Xcoxieuropaeum:0.00298059,KC567179Xcoxieuropaeum:0.007080532,KC567177Xcoxieuropaeum:0.007101402,KC567176Xcoxieuropaeum:0.01058505):0.01169699,AY601624Xdiversicaudatum:0.01008234):0.03013179,(KC567170Xbelmontense:0.003071328,KC567171Xbelmontense:0.003117761,KC567172Xbelmontense:0.0113315):0.0475412):0.02626147,(Xturdetanense_ST90:0.01626145,KC567186Xturdetanense:0.006556627):0.05267931,((Xpseudocoxi_ar95_28S:0.006838469,Xpseudocoxi_ar95_28s_pimpollar:0.004712343):0.04144444,GU549474Xglobosum:0.02482675):0.03342692):0.02217019):0.02572352,(HF546080Xmacroacanthum:0.06855756,HF546081Xmacroacanthum:0.003666105):0.1196972,(KM283422Xindex:0.03701543,(HG969307Xindex:0.002740078,HM921401Xindex:0.003053629,HM921403Xindex:0.003098392,HM921404Xindex:0.002951216,HM921405Xindex:0.002711382,HM921398Xindex:0.002733062,HM921399Xindex:0.00281512,HM921400Xindex:0.002995879,HM921406Xindex:0.002969727,HM921363Xindex:0.002865629,HM921349Xindex:0.002857936,HM921347Xindex:0.003046036,HM921364Xindex:0.002862009,KJ802882Xindex:0.002891182):0.006891408,HM921402Xindex:0.002874062,HM921348Xindex:0.002889663,AY601628Xindex:0.002762637,KJ802881Xindex:0.002835451):0.1467802):0.01640935,(((KJ802878Xcretense:0.002896913,KJ802879Xcretense:0.002757742,KJ802880Xcretense:0.003115469):0.006504573,AY601626Xpyrenaicum:0.007696816):0.04591493,(((KJ956388Xingens:0.01128435,KJ956389Xingens:0.006890237):0.02993821,KF446655Xcastilloi:0.02683624):0.0804229,(EU781538Xdentatum:0.01926217,AY601627Xdentatum:0.002573382):0.07441427):0.03696569):0.02533817):0.04054239,((AY601629Xbasiri:0.003088903,AY601630Xbasiri:0.006703335):0.02815267,AY601631Xcoxi:0.1160377):0.0656656):0.09033786,((((((KP793046Xhunaniense:0.00653243,KP793047Xhunaniense:0.00283917):0.009630446,KP793049Xhunaniense:0.003846925):0.01055145,KP793048Xhunaniense:0.003178081,(KF290564Xhunaniense:0.002958997,EF188840Xhunaniense:0.006818715):0.02257728,EF188839Xhunaniense:0.01709645):0.02063284,EF188841Xhunaniense:0.005493802):0.006409911,EF026090Xhunaniense:0.00425926):0.227211,(((Xturcicum_ST90:0.007183576,KC567185Xturcicum:0.002895173,(Xturcicum_ST149:0.01144495,GU725077Xturcicum:0.002914639):0.007025706):0.08904873,(KC567183Xlupini:0.01091418,HM921352Xlupini:0.003226738):0.257643):0.04504703,(KX062686XspP112016:0.002921631,KX062687XspP112016Z:0.002880596,KX062688XspP112016:0.00755382):0.148925,((Xoleae:0.002906869,1Xoleae:0.002695866):0.009384245,2Xoleae:0.01699993):0.2323662):0.05825266):0.04250731,(((KU680965Xchambersi:0.003021338,KT698206Xchambersi:0.002791606,(KU680967Xchambersi:0.003422473,KT698208Xchambersi:0.003217952):0.006712961,(KU680966Xchambersi:0.002792162,KT698207Xchambersi:0.003048774):0.0198721,KT698205Xchambersi:0.00271673,DQ299512Xchambersi:0.002879693):0.02422227,AY601617Xchambersi:0.003086158):0.006630351,DQ299515Xnaturale:0.003655297):0.1548445,(((KF430801Xelongatum:0.00280284,EF140790Xelongatum:0.003057811,KF430803Xelongatum:0.007541934,KF430802Xelongatum:0.01109198):0.03839509,AY601620Xsavanicola:0.1490262):0.1038284,(AY601618Xelongatum:0.01046726,AY601619Xinsigne:0.04335747):0.1132093):0.1680251,(((ACC89_setariae:0.02816805,AY601621Xsetariae:0.006886162,(DQ299513Xvulgare:0.003088389,DQ299514Xvulgare:0.003156818):0.00684614):0.006747978,(CD1881b_D23_SB611:0.002972709,CD1881a_D23_SB610:0.003098294):0.004604446):0.2177468,AY601622Xradicicola:0.530078

6):0.04618362):0.04980569,(((((Jao25_nemU50_28S:0.06864682,(((AR63_Xadenohysterum:0.0102401,
((AR78_Xadenohysterum:0.007108977,GU725075Xadenohystherum:0.002800571,JAO6_Xadenohyster
um:0.002873474):0.008300178,KC567164Xadenohystherum:0.01532097):0.009532248):0.01635564,K
C567180Xgersoni:0.06748364):0.01536973,(Xhispidum_AR4_U75:0.006559582,((KC567181Xhispidu
m:0.003016161,HM921346Xhispidum:0.01576728):0.005821422,HM921366Xhispidum:0.007512438):0
.005702353):0.02129819):0.01487293):0.0284764,(((XAR16_cohni:0.005832538,KC567173Xcohni:0.0
6167391):0.06427831,(Xmacroagastrum_AR83_28S:0.04154391,Xmacroagastrum_AR82_28S:0.003184
705):0.0287986):0.01966303,GU725074Xhispanum:0.03530113):0.03512933):0.02981779,((GU725073
Xpyrenaicum:0.05183194,((((KX062674Xitalia:0.002748834,KX062675Xitaliae:0.007212043,KX06267
6Xitaliae:0.0110746):0.007183641,KX062677Xitaliae:0.00737537):0.03058964,(KJ802887Xitaliae:0.00
3041553,AY601613Xitaliae:0.007095021):0.00703394,FJ713153Xitaliae:0.002922636):0.006889751,(K
C567182Xitaliae:0.007434854,(HM921350Xitaliae:0.006783837,HM921351Xitaliae:0.01064355):0.010
96678):0.008303045):0.0645919,((Xsphaecocephalum_AR73:0.002894426,AY601615XspYH2004:0.02
295506):0.008436161,GU725076Xsphaerocephalum:0.01381512):0.05608421,((oc3v5_nemk33_28s:0.0
03116804,o3c4_28snemk42:0.02368331):0.007356449,o3v4_nemk32_28s:0.003671222):0.1194872,(KX
062678Xmeridianum:0.01278574,(KX062680Xmeridianum:0.003274739,KX062679Xmeridianum:0.002
906727):0.009399814):0.1115652):0.01046977,((((((HG969310Xvuittenezi:0.002851521,HG329724Xvu
ittenezi:0.002989063):0.01071518,AY601614Xvuittenezi:0.003085331,HG329725Xvuittenezi:0.011324
99,EF614266Xvuittenezi:0.002924437):0.05410665,JQ240273Xgranatum:0.1120447):0.03005452,(KM5
86345Xherakliense:0.002673192,KM586347Xherakliense:0.006356817,KM586350Xherakliense:0.0027
66393,KM586349Xherakliense:0.01111611,KM586348Xherakliense:0.002915444,KM586346Xheraklie
nse:0.002749907,(KM586352Xherakliens:0.002765731,KM586351Xherakliense:0.002841278):0.006779
127):0.03276076):0.02678242,((KJ802883Xisraeliae:0.002772823,(KJ802886Xisraeliae:0.002859783,KJ
802884Xisraeliae:0.01553527):0.0069398,KJ802885Xisraeliae:0.002917488):0.04731659,JN153101Xza
grosense:0.02972649):0.01398796):0.01299207,(KM199690Xbarense:0.002805483,KM199691Xbarense
:0.002869636,KM199692Xbarense:0.01098088):0.06110686):0.01600521):0.01804973):0.02268234,(Xn
uragicum_JAO36:0.01310503,GU725071Xnuragicum:0.002853194,GU725068Xnuragicum:0.00309128
7,GU725072Xnuragicum:0.002882339,GU725065Xnuragicum:0.002676749,GU725067Xnuragicum:0.0
02784975,GU725069Xnuragicum:0.002810864,GU725070Xnuragicum:0.002730231,GU725066Xnuragi
cum:0.00264348,KC567184Xnuragicum:0.003023657):0.09043222,(KX062683Xrobbinsi:0.003237542,
KX062684Xrobbinsi:0.01155838,KX062685Xrobbinsi:0.01779519):0.1260405,((Xconurum_st45_n4_28
S:0.0280716,KX062673Xconurum:0.003042697,KX062671Xconurum:0.003171006):0.01016921,KX06
2672Xconurum:0.006619266):0.2076144):0.07246078,(((ACC86_Xcostaricense:0.003457656,ACC55_X
costaricense:0.007724772):0.0065141,(ACC46_Xcostaricense:0.0032379,ACC61_Xcostaricense:0.00314
5277):0.005635749):0.175697,(Xkrugi_ACC13bulge:0.002848382,CD1880_D23_SA727:0.002922545,
ACC47_Xkrugi:0.003115783,CD1827_D23_Costa_Rica_SA626:0.009055377):0.3868746):0.1571085):
0.03900365):0.08835664,(KP793050Xbrasiliense:0.002915818,AY601616Xbrasiliense:0.003120606):0.
1693326):0.2275644,(((((KT735104XspJ94:0.006435362,(KU680973Xamericanum:0.01273486,DQ285
668Xcitricolum:0.003314652,DQ299490Xcitricolum:0.003667027,DQ299491Xcitricolum:0.003078885,
DQ299492Xcitricolum:0.003320781,DQ299493Xcitricolum:0.003351572,DQ299494Xcitricolum:0.0031
73181,AY601587Xsantos:0.003283617,(KU680976Xamericanum:0.007814606,AY601591Xamericanu
m:0.003407708,AY601590Xpacificum:0.003286916,AY601592Xcalifornicum:0.007595694,KX263172
Xamericanum:0.003508464,KX263171Xamericanum:0.003263264,KX263170Xamericanum:0.00325382
6,KX263168Xamericanum:0.004628973):0.007972524,KU680968Xrivesi:0.003172532,JQ990029Xsant
os:0.003241729,JQ990030Xsantos:0.007579925,(DQ299495Xgeorgianum:0.003348971,DQ299496Xgeo
rgianum:0.003185834,DQ299497Xgeorgianum:0.003352102,DQ299498Xgeorgianum:0.003235841,DQ2
99499Xgeorgianum:0.003403623,DQ299500Xgeorgianum:0.003398529,DQ299501Xgeorgianum:0.0036
15532,DQ299502Xgeorgianum:0.003258894,((DQ299503Xlaevistriatum:0.003579995,DQ299506Xlaevi
striatum:0.003336945,DQ299505Xlaevistriatum:0.003075636):0.008571874,DQ299504Xlaevistriatum:0.
003334766,AY601586XgeorgianumGG14:0.003167837):0.007727165):0.008025192,(KU680974Xameri
canum:0.00830296,KU680975Xamericanum:0.01363283):0.008051609):0.007781286,(AY601588Xrives
i:0.002929522,AY601589Xrivesi:0.00321054,KX263149Xrivesi:0.005254849):0.01199753,KX263160X
californicum:0.008494608,(((KX263144Xcalifornicum:0.003566687,KX263143Xcalifornicum:0.003409
932):0.0128717,KX263145Xamericanum:0.003679637):0.008299483,KX263121Xsp1SAS2016:0.01743
006,KX263142Xsp1SAS2016:0.008168653,KX263122Xsp1SAS2016:0.003663539,KX263137Xsp1SAS
2016:0.003464553,KX263130Xsp1SAS2016:0.003255159,KX263127Xsp1SAS2016:0.003446046,KX2
63126Xsp1SAS2016:0.003572792,KX263125Xsp1SAS2016:0.003251907,KX263129Xsp1SAS2016:0.0
03461594,KX263136Xsp1SAS2016CD472:0.003256601,KX263132Xsp1SAS2016:0.003462778,KX263
131Xsp1SAS2016:0.003484733,KX263120Xsp1SAS2016:0.003070094,KX263119Xsp1SAS2016:0.003
604495,KX263135Xsp1SAS2016:0.008824937,KX263128Xsp1SAS2016:0.003468879,KX263118Xsp1

SAS2016CD471:0.007917756,KX263134Xsp1SAS2016:0.003519737,KX263133Xsp1SAS2016:0.0032
77781,KX263124Xsp1SAS2016:0.003866479,(KX263141Xrivesi:0.02555006,(KX263140Xrivesi:0.003
194725,(KX263139Xrivesi:0.003192127,KX263138Xrivesi:0.003086003):0.008095818):0.008928139,J
Q990042Xparabrevicolle:0.02377114):0.01273136,KX263123Xsp1SAS2016:0.01926001):0.01303607,
KX263152Xamericanum:0.01345979,KX263167Xsp2SAS2016:0.003474594,KX263166Xcalifornicum:
0.003465096,KX263157Xcalifornicum:0.003499569,KX263158Xcalifornicum:0.003299617,KX263156
Xcalifornicum:0.003595803,KX263161Xcalifornicum:0.003365568,KX263159Xcalifornicum:0.0079942
78,KX263151Xamericanum:0.008143963,(KX263165Xcalifornicum:0.003381591,KX263164Xcaliforni
cum:0.003620546,(KX263162Xcalifornicum:0.007933205,KX263163Xcalifornicum:0.008539537):0.00
8163461):0.008110519,KX263150Xamericanum:0.007887072,KX263175Xamericanum:0.003360766,K
X263174Xamericanum:0.003387619,KX263173Xamericanum:0.00349847,KX263169Xamericanum:0.0
04291277,KX263146Xamericanum:0.006550996,KX263154Xcalifornicum:0.005271401,(KX263148Xs
p4SAS2016:0.0126633,KX263147Xsp4SAS2016:0.003792179):0.007833743,(KX263155Xcalifornicum
:0.01386161,KX263153Xcalifornicum:0.006003562):0.01384197,((DQ299507Xfloridae:0.003335271,D
Q299508Xfloridae:0.003201583,DQ299509Xfloridae:0.00322912,DQ299510Xfloridae:0.003320124,DQ
299511Xtarjanense:0.008159372):0.01305344,KP268965Xluci:0.00528157):0.007974577,((((KX263179
Xbrevicolle:0.008427084,(HM163209Xbrevicollum:0.004717069,(HM163211Xlambertii:0.01321722,H
M163210Xinaequale:0.03282091):0.007546471):0.01658546,AY601602Xtaylori:0.003441581,AY60160
3Xtaylori:0.03244493):0.007920069,AY601604Xbrevicollum:0.004713581):0.007962121,KR095280Xb
revicollum:0.003342533,AB635402Xbrevicolle:0.003317517,FM211649Xbrevicollum:0.003349981,AB
675668Xbrevicolle:0.003317107,(AB675667Xbrevicolle:0.003267643,HQ184473Xbrevicollum:0.00347
6912):0.00796688,AB635401Xbrevicolle:0.00332672,KF430800Xbrevicollum:0.005432415,(KP793051
Xbrevicolle:0.003144835,KP793052Xbrevicolle:0.003225959,KP793053XbrevicolleS:0.00320311,KP79
3054Xbrevicolle:0.003171114,KP793055Xbrevicolle:0.008293434):0.02037645,(JN091972Xamericanu
m:0.003262263,AB635403XspJPNHS09:0.003214476):0.02334343):0.008915765,AY601600Xdiffusum
:0.01731165,AY601601Xbrevicollum:0.003159804,AY601605Xbrevicollum:0.003239344):0.02884843)
:0.01206266,((JQ990039Xrivesi:0.00341974,HM921358Xrivesi:0.003526237,HM921357Xrivesi:0.0032
64227,KX263176Xrivesi:0.003652317,JQ990038Xrivesi:0.01867795,JQ990037Xrivesi:0.008016807,KP
268971Xrivesi:0.003306966):0.008547725,AY601593Xthornei:0.008059741,AY601599Xamericanum:0.
008199378):0.008133047,KU680969Xrivesi:0.007729402,KU680972Xrivesi:0.003536231,KU680970Xr
ivesi:0.007319478,JX912150Xrivesi:0.003521999,JX912151Xrivesi:0.003215763,AY601595Xthornei:0.
003058431,AY601598Xutahense:0.003277473,AY601597Xincognitum:0.01027643,AY601596Xbricole
nsis:0.007460168,KU680971Xrivesi:0.008403283,AY601594Xbricolensis:0.006554233,AY210845Xrive
si:0.003215432,KX263178Xsp1SAS2016:0.003375793,KX263177Xsp1SAS2016:0.003388056,JX91214
9Xrivesi:0.00336533):0.3035088,(KP268966Xmadeirense:0.06862255,JQ990041Xparatenuicutis:0.1370
547):0.2292415):0.08710914,((((((((Xincertum_AR7_28S:0.002957921,JQ990031Xincertum:0.0071592
84,KU250157Xpenevi:0.003084124,Xincertum_AR30_28S:0.007444707):0.00853973,(KP268964Xince
rtum:0.003228476,Xincertum_AR104_28S:0.003298765):0.007081468):0.01222043,((KX263184Xpacht
aicum:0.007887806,KP268968XpachtaicumA:0.008751059):0.007773875,((KX263182Xpachtaicum:0.0
03195433,JQ990033Xpachtaicum:0.007477231,AY601606Xpachtaicum:0.01143559,(HM921395Xpacht
aicum:0.002827565,HM921354Xpachtaicum:0.002854589):0.007459037):0.00720391,(HM921391Xpac
htaicum:0.003043786,HM921365Xpachtaicum:0.002983916):0.0070349,HM921353Xpachtaicum:0.002
989693,(HM921393Xpachtaicumn:0.008444002,KX263181Xpachtaicum:0.008470818):0.007131691,K
X062681Xpachtaicum:0.0360302):0.007685752,KJ802888Xpachtaicum:0.003029692,KU250155Xpacht
aicum:0.003056691,(HM921394Xpachtaicum:0.002959108,HM921355Xpachtaicum:0.002944973):0.00
7060525,(HM921396Xpachtaicum:0.003125813,HM921356Xpachtaicumn:0.002791141):0.007277168,
HM921389Xpachtaicum:0.003058361,HM921392Xpachtaicum:0.00313159,KX062682Xpachtaicum:0.0
0310957,HM921390Xpachtaicum:0.002962382,HM921397Xpachtaicum:0.003438817,KX263183Xpach
taicum:0.01691646):0.03309398):0.06695687,((Xparapchydermum_28s_AR35:0.004131413,KP268969
XparapachydermumA:0.009024892):0.01490168,JQ990034Xparapachydermum:0.003298655,JQ990035
Xparapachydermum:0.009939527,JQ990036Xparapachydermum:0.01522161,KP268970Xparapachyder
mum:0.01987149):0.05080349):0.01860966,KP268955Xastaregiense:0.04459825):0.02084488,((KX263
186Xsp3SAS2016:0.002869592,KX263185Xsp3SAS2016:0.003228588):0.02561219,AY601607Xpacht
aicum:0.01067054):0.07857834):0.02784109,(KP268956Xplesiopachtaicum:0.002932295,KP268958Xpl
esiopachtaicum:0.006116783,KP268957Xplesiopachtaicum:0.003094008):0.04641115):0.1594515,AY60
1608Xpachydermum:0.1917492):0.0880208,((KP268959Xvallense:0.003322192,KP268960Xvallense:0.
002914661,KP268961Xvallense:0.002971419):0.2524367,(((KU250151Xbrowni:0.003331673,KU25015
3Xbrowni:0.003298403,KU250152Xbrowni:0.003353764,KU250154Xbrowni:0.002873514):0.1334222,
KU250156Xparasimile:0.04548806):0.1835146,((KX263180Xsimile:0.01013382,JQ780368Xsimile:0.00
3485115,JQ780370Xsimile:0.003438799,JQ780369Xsimile:0.00332326,(KJ802889Xsimile:0.008091218

,KJ802890Xsimile:0.003643342):0.007680816):0.008017043,AY601609Xsimile:0.00573311):0.291093
9):0.05776355):0.06207661):0.06219213):0.06410484,(KP268962Xbrevisicum:0.00752585,AY601610X
brevisicum:0.00380798):0.2074632,((KP268963Xduriense:0.01619653,JQ990032Xduriense:0.00391128
2):0.0239138,(KP268967Xopisthohysterum:0.003471414,JQ990040Xopisthohysterum:0.003377384):0.0
588685):0.1298855):0.3141723):0.1026592,((((((((KJ802862Lcloselongatus:0.003582598,KJ802863Lclo
selongatus:0.003308062,KJ802866Lcloselongatus:0.003278432,KJ802864Lcloselongatuscret3:0.003102
961,KJ802865Lcloselongatuscret4:0.003223302):0.0487295,(((KJ802867Lcretensis:0.007615027,KJ802
869Lcretensis:0.003125611,KJ802868Lcretensis:0.01201473):0.04914386,KF242334Lsp4SAS2014CD1
117:0.01429875):0.02700243,((KJ802870Lpseudoelongatuscret9:0.003565514,KJ802872Lpseudoelongat
uscret11:0.003248489,KJ802871Lpseudoelongatus:0.003372432,KJ802873Lpseudoelongatus:0.0079610
64):0.03146281,((KJ802874Lmoesicus:0.003539879,HM447029LmoesicusVrhpolje:0.00340547,(KJ802
876Lmoesicus:0.002967866,KJ802876Lmoesicuscret15:0.003078788):0.009017106,(KJ802875Lmoesic
us:0.003837743,KJ802875Lmoesicuscret14:0.003898937):0.009073652):0.007362803,KP222294Liranic
usLi90:0.003779091):0.01828091):0.02979171):0.02466635):0.03446937,(KF242335Lsp3SAS2014CD1
112:0.02987451,(((KX062667LeuonymusR30voucherLEP12:0.0176736,KR152295LperangustusSFR_1:
0.03712241):0.02079396,KF242333LeuonymusCD1118:0.003216005,KF242331LeuonymusCD1086:0.0
0324013,KF242332LeuonymusCD1130:0.007377515,AY601573Leuonymus:0.01211607):0.06297334,(
KU747173LspPC2016ES1:0.00331218,KU747174LspPC2016AA14:0.003473729):0.0662315):0.051682
01,(KT755457LattenuatusFK329:0.003373238,AY601572Lattenuatus:0.003256726,KR911851Lattenuat
usFK320:0.003301811):0.07526219,((KX398055Lkuiperi:0.003500265,KX398056LkuiperiA:0.0032799
28):0.08832632,(((KX062668LglycinesR15voucherLGP11:0.01551326,(KX062670LglycinesR25vouche
rLGP4:0.01278195,KX062669Lglycinesn72voucherLGP3:0.003410406):0.01069121):0.09172943,AY60
1575Ledmundsi:0.1976432):0.03559121,(((KF242330Lsp1SAS2014CD833:0.06704617,KF242328Lsp6
SAS2014CD876:0.05318173):0.01717449,KF242329Lsp2SAS2014CD897:0.01975365):0.05943685,AY
601574Lathesinus:0.06230926):0.105719):0.02522207):0.01947215,AY601571Lapulus:0.1422129,((((K
T308861LvallensisN35:0.00345233,KT308862LvallensisA84:0.01860133):0.06951055,JX445116Lrubi8
96:0.024928):0.06159107,(((((((JX445105LiuglandisD03:0.003326476,JX445104LiuglandisH33:0.00354
8161):0.1440387,((JX445106LbaeticusC80:0.01324581,JX445107LbaeticusB41:0.03794419):0.1565039
,(KT308856LmacrodorusV33:0.03284128,KT308855LmacrodorusV4:0.2971497):0.1100558):0.0507302
5):0.04330459,JX445108LfasciatusA15:0.1566415):0.04911173,((KT308871LoleaeQ56:0.006753187,J
X445103LoleaeE77:0.003671204):0.1175679,(((KJ802877Lorientaliscret16:0.003305967,GQ988722Lor
ientalisfromIran:0.003375695,KP406932LorientalisCD1379:0.003336595,KP406934LorientalisCD1437:
0.003240261,KP406933LorientalisN10:0.00757856,KP406931LorientalisP28:0.003267045):0.00806610
5,GU001823Lorientalis:0.004343787):0.02513847,(KF242336Landalusicus868:0.007124306,JX445102
Landalusicus600:0.003338464,JX445101LandalusicusG65:0.008307437):0.1004199):0.07056293):0.032
06194):0.02364023,((KT308872LvineacolaL73:0.008455557,(JX445110LvineacolaF23:0.003509501,JX
445109LvineacolaC79:0.01854536,JX445111LvineacolaA54:0.0221108):0.007423793):0.007575544,KT
308873LvineacolaL99:0.01497778):0.1006615,(((((KT308857LonubensisL47:0.003300356,KT308858L
onubensisw31:0.003305645):0.09216411,AY601581Lgoodeyi:0.04771924,JX445114LcrataegiC92:0.104
3821):0.01522392,KT308869LlusitanicusK2:0.1065815):0.02048505,((KT308870LmagnusW22:0.00887
6746,JX445112LmagnusB90:0.003196381,HM921361Lmagnusnem229:0.003413013,JX445113Lmagnu
sD62:0.003321537,HM921362Lmagnusnem230:0.01319084):0.03416736,((KT308875LvinearumO28:0.
003653389,KT308876LvinearumU69:0.00330074,KT308874LvinearumN74:0.02493213):0.01325148,K
T308877LvinearumV18:0.007949388):0.05237889):0.0316592):0.05103082,((KT308859LsilvestrisX11:
0.02713136,KT308860LsilvestrisL58:0.003602228):0.0204471,((KT308863LwicuoleaU50:0.003269559,
KT308864Lwicuoleaw16:0.003851687,KT308866Lwicuoleax9:0.007814743):0.009345505,KT308865L
wicuoleaw81:0.02693979):0.05348762):0.102865):0.04905618):0.1178428,(((((KT308868Lintermediusx
4:0.02411714,JX445117Lintermedius642:0.003637553):0.01360006,(KF242312LintermediusCD1122:0.
01361952,KF242311LintermediusCD1085:0.009297027):0.008898998):0.01381613,AY601577Lpiceicol
a:0.009610942):0.02993847,EF538754Luroshis:0.05264615,(KF242316LartemisiaeCD1144:0.00389909
2,(KF242315LartemisiaeCD1146:0.003504422,(KF242314LartemisiaeCD1127:0.003494503,KF242313
LartemisiaeCD1115:0.00342331):0.006548181):0.007113265):0.1018309,((KF242310LelongatusCD114
5:0.006461803,HM447032LelongatusMaribor:0.02994309):0.01101575,KF242299LelongatusCD1205:0.
003625271,KF242306LelongatusCD1114:0.003726664,KF242304LelongatusCD1132:0.008034835,KF2
42298LelongatusCD1125:0.003523306,KF242297LelongatusCD1126:0.00371051,AY601578Lelongatus
:0.003581461,KF242296LelongatusCD1123:0.003157925,KF242294LelongatusCD1345:0.003443101,K
F242309Lelongatus591:0.003474864,KF242308LelongatusCD1139:0.003578172,KF242307Lelongatus
CD1141:0.00325382,KF242305LelongatusCD1135:0.003489252,KF242303LelongatusCD1140:0.00362
4155,KF242301LelongatusCD1116:0.003477086,KF242302LelongatusCD1290:0.003692075,KF242295
LelongatusCD8541:0.00823294,KF242300LelongatusCD1134:0.003724622):0.07344762):0.0728459,(((

KF292307Laetnaeus122:0.003400742,(KF242324LaetnaeusCD1138:0.005175095,KF242323LaetnaeusC
D1121:0.003578463):0.01163334,KF242319LaetnaeusCD1143:0.003435249,KF242318LaetnaeusCD11
11:0.003371112,KF242322LaetnaeusCD1142:0.003267824,KF242321LaetnaeusCD1129:0.003294279,K
F242320LaetnaeusCD1108:0.003510548,(KF242327LleptocephalusCD1119:0.003683088,KF242326Lle
ptocephalusCD1136:0.003537541,DQ364600Lleptocephalus:0.003222856,AY601580Lleptocephalus:0.0
1796537,KF242325LleptocephalusCD1131:0.003572621):0.02632801,KC357770Laetnaeus:0.00318727
7,KC357771Laetnaeus:0.007936383):0.117229,((KF242317LdistinctusCD11281:0.003258941,EF65453
9Ldistinctus:0.00333031):0.07995321,(DQ364599Ljuvenilis:0.03619436,AY601579Ljuvenilis:0.004285
81):0.05768391):0.02979332):0.020676):0.05076212):0.04755072,(((KT308867Lalvegusw86:0.0032569
45,JX445115LalvegusE76:0.01309763):0.008264462,HM921360Lalvegusnem228:0.00356605):0.00794
3254,HM921359Lalvegusnem227:0.01459797):0.1770637):0.02905386):0.03185859,(KT308852Lindalu
sM44:0.003262541,KT308853LindalusM40:0.003290395,KT308854LindalusW27:0.01236178):0.14545
17):0.02559581,AB477069LspDLP003for:0.1146051,AY601576Lbreviannulatus:0.1740047,((KT75545
8LdanuviiFK305:0.003485914,KU507542LdanuviiFK050:0.003551398):0.08147321,AY601570Larthen
sis:0.09999129):0.06128098):0.02039372):0.03555576,AY494715Lamericanum:0.1571725):0.09554393
,HQ735098LspITDL2011:0.5244749):0.0791984,((((KX062664Lafricanusz18voucherAFP3:0.00330733
3,(KX062665Lafricanusz17voucherAFP1:0.007606657,KX062666LafricanusR29voucherAFP12:0.0079
81454):0.007867802,KF242339LafricanusCD832:0.007854345):0.007692453,KF242341LafricanusCD1
511:0.01687229,KF242340LafricanusCD831:0.007529252,KF242337LafricanusCD1512:0.007900467,K
F242338LafricanusCD1301:0.003241407,KP711809Lafricanus:0.003541795):0.007581526,AY601583L
africanus:0.005701368):0.2389623,(((KJ427790ParaLrex:0.003314167,KJ427792ParaLrex:0.003085272,
AY601582ParaLspYH2004:0.003219823,KJ427793ParaLrex:0.008202933,KJ427791ParaLrexFK:0.003
18183):0.06812293,JN032587ParaLiranicus:0.1696057):0.03006759,(KF412826ParaLmaximus:0.04732
201,((EU026155ParaLlitoralis:0.03976043,EU026156ParaLparamaximus:0.05553636):0.0312815,JQ673
403ParaLplesioepimikis:0.09164458):0.03882474):0.02657352):0.2234287):0.0512661):0.08355667,(((((
KR351254LasiaticusG80:0.003169228,KR351255Lasiaticus6_59:0.01260627,KR351256Lasiaticus59_T
7:0.02461584):0.1669243,KJ873104LhangzhouensisstrainNB004:0.2582426):0.1608868,((KJ741239Lsp
JH2014strain82461:0.003246717,KJ741240LspJH2014strain82462:0.003340132,KJ741241LspJH2014st
rain82463:0.003306685):0.1224453,AY601585Lcamelliae:0.07951699):0.1878883):0.2149196,(HQ8222
66LhenanusHZ001:0.005945571,HQ822267LhenanusFY028:0.003800306):0.5231072):0.08174767,(((K
F280150LspLTD23:0.07019045,(KF242343Lsp5SAS2014CD5062:0.003372347,KF242342Lsp5SAS201
4CD5061:0.01300531):0.06028677,AY601584Ldiadecturus:0.1208307):0.02804973,KF552069LjonesiL
5:0.009726248):0.2849681,KF270638LspLSD253:0.3666457):0.1760201):0.1122045):0.04238591,(((((((
(KJ567473Lcaespiticola64L28SlargesubunitribosomalRNA:0.003458839,KJ567472Lcaespiticola30L28S
largesubunitribosomalRNA:0.003177847,HM447030LcaespiticolaBrdo:0.02820485):0.01421556,KF242
344LcaespiticolaCD1109:0.03106662):0.01683151,AY601567Lcaespiticola:0.03660468):0.08563565,((F
R775761LspFDL2011partial28SrRNAindividual58:0.003204479,FR775762LspFDL2011partial28SrRN
Aindividual59:0.003193417):0.1222478,(KF242347LlignosusCD1120:0.005686124,(KF242346Llignosu
sCD1133:0.00308737,KF242345LlignosusCD1124:0.003430614):0.006876444):0.1545625):0.03378825
):0.02733422,((EF538750Lpoessneckensis:0.002981865,EF538751Lpoessneckensis:0.007858738):0.096
40575,(JN631811LcarniolensisKrmacina:0.003334906,JN631812LcarniolensisDrasici:0.003337351):0.1
717861):0.051979):0.03336877,(((JN627414LhelveticusCH28SlargesubunitribosomalRNA:0.003349321
,JN627415LhelveticusMOR28SlargesubunitribosomalRNA:0.003218136,EF538753Lhelveticus:0.00326
6161,HM447031LhelveticusTrskagora:0.007986889):0.008138024,AY601566Lhelveticus:0.01843099):0
.03663016,(AY601565Lmacrosoma:0.003313557,EF538752Lmacrosoma:0.02629073):0.03997731):0.06
601085):0.185048,(JQ240274Lpisi:0.003718595,JQ240274LpisiH56:0.003540132,AY601568Llatocepha
lus:0.013543,AY601569Llatocephalus:0.007907812):0.4611016):0.03374844,JN032584ParaLbikanerens
is:0.3084156):0.07130956):0.1468785):0.1586605,(KX136868LlaevicapitatusP53:0.003016028,KX1368
65LlaevicapitatusO36:0.00317735,KX136866LlaevicapitatusO48:0.003008099,KX136867Llaevicapitatu
sP52:0.003122109):0.3158108):2.206304);

Supporting Information Figures (Figs S1-S3)

Figure S1. Phylogenetic relationships within Longidoridae. Bayesian 50% majority rule consensus tree as inferred from the analysis of the partial *coxI* sequence alignment under a TrN + I+ G model. Posterior probabilities more than 0.7 are given for appropriate clades. New sequences obtained in this study are in bold.

Figure S2. Phylogenetic relationships within Longidoridae. Bayesian 50% majority rule consensus tree as inferred from the analysis of the first and second nucleotide from each codon from the partial *coxI* sequence alignment under a TVM + I+ G model. Posterior probabilities more than 0.7 are given for appropriate clades. New sequences obtained in this study are in bold.

Figure S3. Phylogenetic relationships within Longidoridae. Bayesian 50% majority rule consensus tree as inferred from the analysis of the D2-D3 region alignment under a GTR + I + G model. Posterior probabilities more than 0.7 are given for appropriate clades. New sequences obtained in this study are in bold.

coxI

FIGURE S1

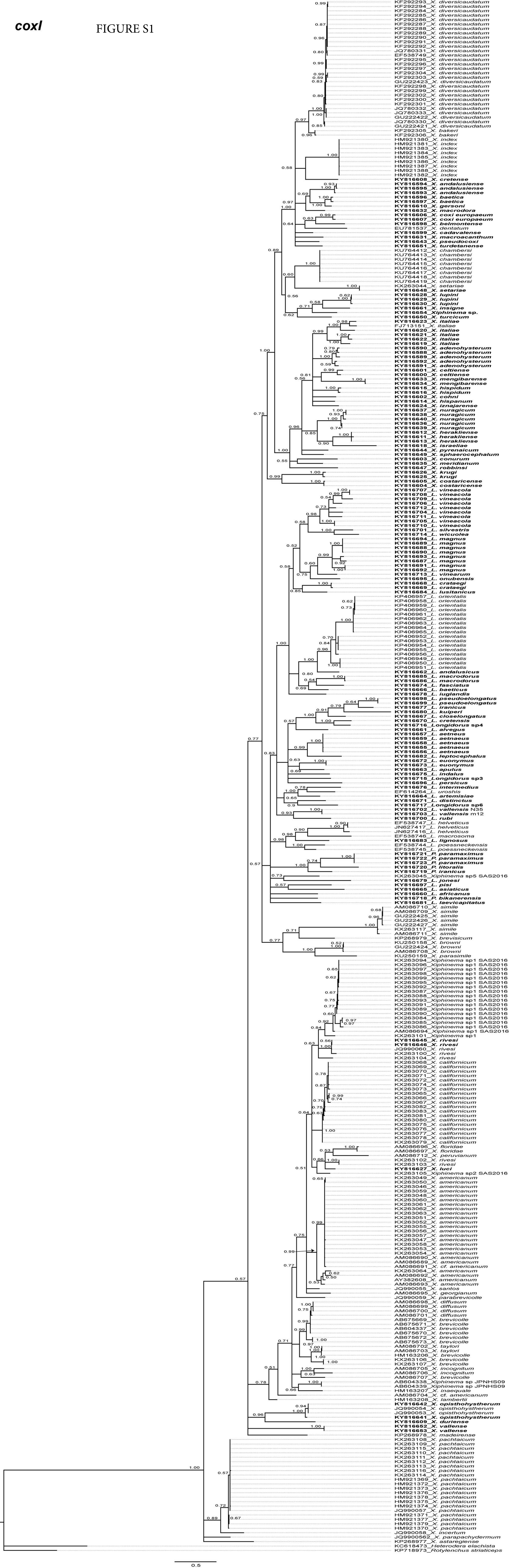

coxI FIGURE S2

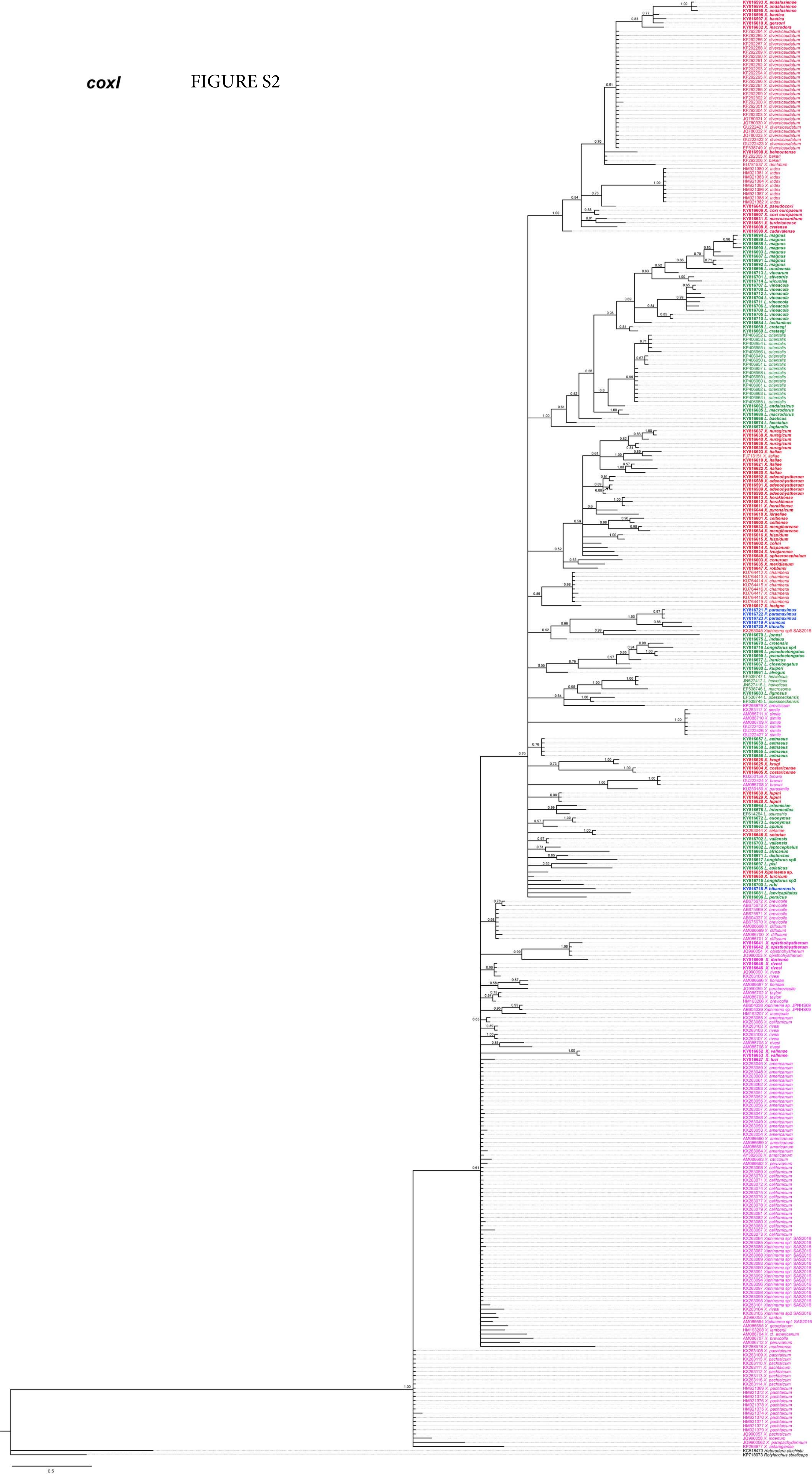

FIGURE S3

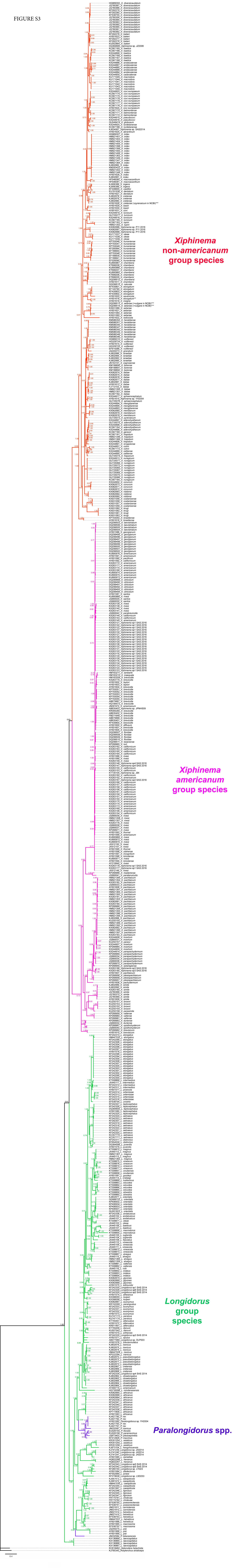

Xiphinema non-americanum group species
Xiphinema americanum group species
Longidorus group species
Paralongidorus spp.
